# Coupling the MAPK Slt2/ERK1 Pathway and IRE1-driven UPR Through Transcription Factor Rlm1/MEF2

**DOI:** 10.21203/rs.3.rs-7292507/v1

**Published:** 2025-12-11

**Authors:** Anish Chakraborty, Saswata Chakrabarty, Jagadeesh Kumar Uppala, Kimberly Ann Mayer, Anna J Evans, Jasmine George, Chandrima Ghosh, Ritisha Dey, An Phu Tran Nguyen, Nadege Gouignard, Pradeep Chaluvally-Raghavan, Madhusudan Dey

**Affiliations:** 1 Department of Biological Sciences, UW-Milwaukee, WI-53211; 2 Department of Obstetrics and Gynecology, Medical College of Wisconsin, Milwaukee, WI-53226.

**Keywords:** Protein kinase, Endoplasmic reticulum stress (ER stress), Unfolded protein response (UPR), Mitogen-activated protein kinase (MAPK), Extracellular-signal-regulated kinase (ERK), IRE1 (Inositol requiring enzyme 1), Slt2, *HAC1* mRNA

## Abstract

Unfolded protein response (UPR) is a conserved cellular strategy that enhances the protein folding capacity of cells under stress conditions. In *Saccharomyces cerevisiae,* the dual kinase RNase IRE1 initiates the UPR by catalyzing the cytosolic splicing of *HAC1* mRNA, a process conserved in humans where IRE1 splices *XBP1* mRNA. The spliced *HAC1/XBP1* mRNA yields a transcription factor that upregulates the expression of protein-folding enzymes and chaperones, thereby boosting the cell’s ability to cope with unfolded proteins. Our study demonstrates that the UPR involves two distinct phases. The early phase operates predominantly through the canonical IRE1 signaling pathway, while the later phase involves additional regulation by the MAP kinase Slt2 or its human ortholog ERK1/ERK2/ERK5 and the downstream target the MADS-box transcription factor Rlm1 (an ortholog of human MEF2C). We further show that Slt2 promotes IRE1 expression through Rlm1. Together, these findings reveal a previously unrecognized crosstalk between the MAPK and IRE1-mediated arm of the UPR.

## INTRODUCTION

Mitogen-activated protein kinases (MAPKs) represent a large family of kinases, which are activated by a diverse set of extracellular and intracellular stimuli including growth factors, cytokines, and a variety of extra- and intra-cellular stresses [1, 2]. In humans, the MAPK family is composed of four major subgroups: (1) extracellular signal-regulated kinase (ERK) [3, 4]; (2) big MAP kinase (BMK) [5]; (3) Jun N-terminal kinase (JNK) [6–8]; and (4) the p38 kinase [9–11]. Members of ERK (e.g., ERK1 and ERK2) and BMK families are primarily activated by growth factors and cytokines, whereas the p38 and JNK families respond primarily to intra- and extracellular stresses, thus classified as the stress-activated protein kinases (SAPK) [12].

Activation of MAPK typically occurs through a conserved three-tiered kinase cascade (MAP3K → MAP2K → MAPK), in which an upstream MAP3K phosphorylates and activates a MAP2K, which then phosphorylates and activates a terminal MAPK. For example, an upstream Raf kinase (MAP3K) phosphorylates and activates MEK1 or MEK2 (MAP2K), which phosphorylates and activates ERK1 or ERK2 (MAPK). Like ERK1/2, BMK1 is activated upon phosphorylation by upstream MAP2K (MEK5α or MEK5β) that is activated upon phosphorylation by MAP3K (MEKK2 or MEKK3) [13]. JNK isoforms are activated by upstream MAP2K kinases MKK4 and MKK7[14], whereas p38 isoforms are activated by kinases MKK3 and MKK6[15]. The active MAPK is often associated with a scaffold protein that helps integrate the signaling circuits and regulates a diverse set of cellular functions, including cell growth, proliferation, and differentiation [16].

In the budding yeast *Saccharomyces cerevisiae*, five distinct MAPKs (Fus3, Kss1, Hog1, Slt2 and Smk1) mediate various cellular signals [17]. Among these, the Hog1 and Slt2 kinases are known as SAPKs. Hog1 is the yeast orthologue of human p38[10, 18] and is regulated by two MAP3K kinases (Ste11 and Ssk2) and a single MAP2K (Pbs2). Slt2, the yeast orthologue of human BMK1, also known as ERK5 [19], is activated by MAP3K (Bck1) and two redundant MAP2Ks (Mkk1 and Mkk2) [20, 21]. The Hog pathway is activated by rapid changes in environmental osmolarity [18], whereas the Slt2 pathway responds to the cell wall stress [17, 22], high temperature [23], osmotic shock [24] and polarized growth[21], as well as stresses resulting from the accumulation of unfolded proteins within the endoplasmic reticulum (ER) [25].

Within the ER, most secreted and membrane proteins fold and mature. When this process fails, unfolded proteins accumulate in the ER, causing an imbalance in ER proteostasis, a state known as the ER stress. In response, cells trigger the unfolded protein response (UPR), an adaptive strategy to restore the ER proteostasis. UPR is controlled by three major ER-resident sensors: dual kinase RNase IRE1 (inositol requiring enzyme 1) [26–28], protein kinase PERK (Protein kinase R-like endoplasmic reticulum kinase) [29] and transcription factor ATF6 (Activating transcription factor 6) [30]. IRE1 is the conserved sensor from yeast to humans, which initiates the UPR by cleaving *HAC1* mRNA in yeast cells or *XBP1* mRNA in metazoan cells. The cleaved *HAC1* mRNAs are ligated by tRNA ligase[31], whereas RTCB ligates *XBP1* mRNAs [32]. The spliced *XBP1* or *HAC1* mRNA yields a transcription factor that upregulates the expression of protein folding enzymes and chaperones to manage unfolded proteins. When ER stress is severe, IRE1 broadens its substrate specificity and cleaves ER-associated mRNAs in a process known as the regulated IRE1 dependent degradation (RIDD) pathway [33, 34].

Although the role of IRE1 in UPR is well characterized, the precise function of Slt2 in this process remains unclear. In 2005, Chen et al. proposed that Slt2 and IRE1 kinases activate parallel signaling pathways based on their findings that splicing of *HAC1* mRNA in *slt2Δ* strains was comparable with the wild type strain when grown in the presence of an ER stressor for one hour[35]. A limitation of their study is that cells were stressed only for one hour, which might not be adequate to fully elicit the ER stress response. In 2016, Rousseau and Bertolotti claimed that Slt2 plays a role in the assembly of a functional 26S proteasome based on their findings that chaperones for the 26S proteasome – such as Adc17, Hsm3, Nas6, Nas2 and Rpn14 – are expressed at low levels in ER-stressed *slt2Δ* cells[36]. However, yeast strains lacking Nas6, Hsm3, Adc17, Rpn14 grow well under an ER stress condition, raising concerns about their precise role in UPR. In this study, we provide evidence that Slt2 and its target Rlm1 act in coordination with the IRE1 pathway to mitigate ER stress. Specifically, we show that Rlm1 promotes the expression of IRE1 during the adaptive state of UPR. We also show that human MAPK ERK1 and MEF2C can functionally substitute for yeast MAPK Slt2 and Rlm1, respectively. Notably, the activation pattern of ERK1 in human cells mirrors that of Slt2 in yeast cells. Taken together, these findings uncover a previously unrecognized crosstalk between the MAPK Slt2/ERK1 and IRE1-mediated UPR signaling pathway.

## RESULTS:

### 1. Slt2 promotes cytosolic splicing of *HAC1* mRNA.

The budding yeast *S. cerevisiae* contains five MAPKs on five functionally distinct cascades[37] (Fig. S1A). To investigate the role of these MAP kinases in the ER response, we compared growths of wild-type (WT) yeast with its isogenic strains, each lacking a single MAP3K, MAP2K or MAPK, on the medium containing an ER stressor, either tunicamycin or DTT. Tunicamycin inhibits N-linked glycosylation, while DTT disrupts disulfide bond formation in proteins, resulting in the accumulation of unfolded proteins inside the ER lumen and triggering ER stress. Tunicamycin was primarily used in the solid medium over an extended period, mainly due to its greater stability compared to the rapidly oxidizing DTT.

**Figure 1:**
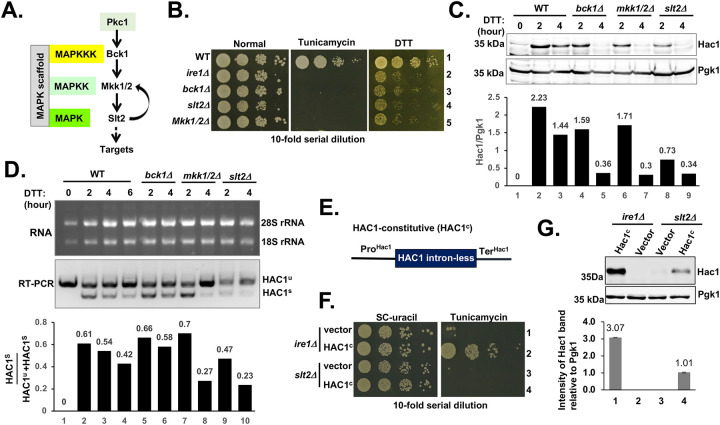
*HAC1* mRNA splicing is reduced in the *slt2Δ* strain. **(A)** The MAPK Slt2 signaling pathway in yeast *S. cerevisiae*. **(B)** The indicated yeast strains were serially diluted, spotted and grown on the YEPD medium and the same medium containing tunicamycin (0.2 μg/ml) or DTT (1 mM) at 30°C for 48 hours. **(C)** The indicated yeast strains were grown in the YEPD medium until they reached the OD_600_ value ~0.5–0.6. Cells were then exposed to DTT (5 mM) and harvested after 2 and 4 hours. WCEs were prepared and subjected to Western blot analysis using antibodies specific to Hac1 and Pgk1 proteins. The intensities of Hac1 and Pgk1 protein bands were quantified using the ImageJ software. A representative result from three independent experiments is shown, with other data in the Fig. S3. **(D)** The indicated yeast strains were grown in the YEPD medium until they reached the OD_600_ value ~0.5–0.6. Cells were then exposed to DTT (5 mM) and harvested after 2 and 4 hours. Total RNA was extracted and subjected to RT-PCR analysis to detect between the spliced (HAC1^s^) and un-spliced (HAC1^u^) *HAC1* mRNAs. The intensities of HAC1 mRNAs were quantified using the ImageJ software. A representative result from three independent experiments is shown with other data in the Fig. S4. **(E)** The schematic diagram of the constitutive *HAC1* allele (HAC1^c^). The intronic sequence from the *HAC1* open reading frame was removed. **(F)** The indicated yeast strains containing a vector plasmid or the same vector bearing a constitutive *HAC1* allele (HAC1^c^) were grown, serially diluted, spotted and grown on the YEPD and the same medium containing tunicamycin at 30°C for 48 hours. **(G)** WCEs were prepared from yeast strains shown in the panel (**F**) and subjected to Western blot analysis using antibodies specific to Hac1 and Pgk1 proteins. (Lower panel). The bar diagram depicts the relative intensities of Hac1 protein bands.

On the medium containing tunicamycin (0.2 μg/ml), yeast cells lacking the kinase Ste20 (MAP4K), Ste11 (MAP3K), Ste7 (MAP2K), Fus3 (MAPK) or Kss1 (MAPK) grew comparably to isogenic WT cells (Fig. S1B). On the same tunicamycin medium, yeast cells lacking the kinase Ssk1, Ssk2, Pbs2 or Hog1 showed slower growth compared to WT (Fig. S1B), whereas cells lacking the kinase Bck1, Mkk1/2 (both Mkk1 and its paralog Mkk2) or Slt2 (Fig. 1A) failed to grow (Fig. 1B), like cells lacking ER-stress sensor IRE1 (Fig. 1B and Fig. S1B). Similarly, medium containing DTT inhibited growth of yeast cells lacking the kinase Bck1, Mkk1/2 or Slt2 (Fig. 1B). Thus, the sensitivity to tunicamycin or DTT indicates that, in addition to the classic Ire1-mediated UPR pathway, both Slt2 and Hog1 pathways are likely to be involved in the ER stress response.

These findings confirmed the earlier studies conducted by Chen et al. (2005), who showed that *bck1Δ*, *mkk1/2Δ* and *slt2Δ* strains were severely sensitive to tunicamycin [35]. We also confirmed Chen et al.’s results that the *slt2Δire1Δ* double mutant was more sensitive to tunicamycin than either single mutant (Figs. S2A and S2B). Additionally, they observed that Hac1 protein levels were comparable in WT and *slt2Δ* strains after one hour of tunicamycin treatment. Together, these results, along with the reduced fitness of the *slt2Δire1Δ* double mutant strain, suggest a negative genetic interaction between IRE1 and Slt2 pathways, with each pathway contributing independently to the ER stress response. However, their experiments did not define a critical threshold at which the decline in cellular fitness becomes prominent. Here, we hypothesized that a one-hour tunicamycin exposure may not be sufficient to reach this threshold while adequately eliciting the ER stress response and replicating the sensitivity to tunicamycin observed on the solid medium. To test this, we treated cells with tunicamycin or DTT treatment for at least 2 hours and investigated the involvement of MAP kinases in the IRE1-mediated ER stress response by comparing splicing of *HAC1* mRNA and expressions of Hac1 protein in *bck1Δ*, *mkk1/2Δ* and *slt2Δ* strains (Fig. 1C).

The yeast strains were grown in a liquid YEPD medium till the OD_600_ value reached to 0.5–0.6. Cells were then treated with tunicamycin or DTT for 2- and/or 4-hours hours. Tunicamycin or DTT-stressed cells were harvested and whole cell extracts (WCEs) were prepared. WCEs were utilized for Western blot analysis to examine the Hac1 protein expression, while reverse transcriptase (RT)-PCR analysis was conducted to assess the *HAC1* mRNA splicing. In cells treated with DTT for 2 hours, Hac1 protein levels were reduced in *bck1Δ* (~30%), *mkk1/2Δ* (~25%) and *slt2Δ* (~70%) cells compared to WT (Fig. 1C, lanes 4, 6 and 8). This reduction became more prominent after 4-hours of DTT treatment, reaching almost ~75% in all three mutants (Fig. 1C, lanes 5, 7 and 9). Similarly, following a treatment with tunicamycin for 2 hours, Hac1 protein levels were reduced 23.5% in *bck1Δ*, 46.45% in *mkk1/2Δ* and 63.7% in *slt2Δ* cells (Figs. S3A and S3B).

The observed reduction of Hac1 protein levels correlated with reduced splicing of *HAC1* mRNA. Specifically, in cells treated with DTT for 4 hours, *HAC1* mRNA splicing was reduced by 22.8% in *bck1Δ*, 14.6% in *mkk1/2Δ* and 46.7% in *slt2Δ* cells as compared WT cells (Fig. 1D and Fig. S4A). A similar trend was observed following the tunicamycin treatment for 2 hours, *HAC1* mRNA splicing was reduced in *bck1Δ*, *mkk1/2Δ* and *slt2Δ* strains (Fig. S3B). These reductions in *HAC1* mRNA splicing and Hac1 protein expressions, together with severe sensitivity to tunicamycin at a low concentration (0.2 μg/ml), suggest that MAP kinase Slt2 and its upstream kinase regulators contribute to folding of ER client proteins by directly influencing the Hac1 protein expression.

To test whether Slt2 kinase influences translation from the spliced *HAC1* mRNA or affects Hac1 protein abundance, we investigated Hac1 protein expression from an intron-less *HAC1* mRNA in the *slt2Δ* strains. To do so, we constructed a plasmid in which the intronic sequence of the *HAC1* ORF was removed. This engineered plasmid was designated as the HAC1-constitutive (HAC1^c^) (Fig. 1E). The HAC1^c^ plasmid was introduced into *ire1Δ* and *slt2Δ* strains. As expected, the *ire1Δ* strain containing the HAC1^c^ plasmid grew on tunicamycin-containing medium (Fig. 1F, row 2), correlating with the expression of Hac1 protein (Fig. 1G, lane 1). In contrast, the *slt2Δ* strain harboring the HAC1^c^ plasmid did not grow on tunicamycin-containing medium (Fig. 1F, row 4). Interestingly, we observed that Hac1 protein was expressed from the HAC1^c^ plasmid in the *slt2Δ* strain (Fig. 1G, lane 4), although at a 3-fold lower compared to the *ire1Δ* strain (Fig. 1G, lower panel). These results suggest that Slt2 plays a role in maintaining the Hac1 protein level and is consistent with the earlier studies that showed that the Hac1-meditaed LacZ expression was lower in the *slt2Δ* strain [35]. Together, these data indicate that both MAPK Slt2 contribute to Hac1 protein expression from the spliced *HAC1* mRNA and/or influence the Hac1 protein abundance. Additionally, these data indicate that Hac1 protein alone is not sufficient to promote cell growth under conditions of ER stress.

### 2. MAP kinase Slt2 is activated during ER stress independent of IRE1.

**Figure 2:**
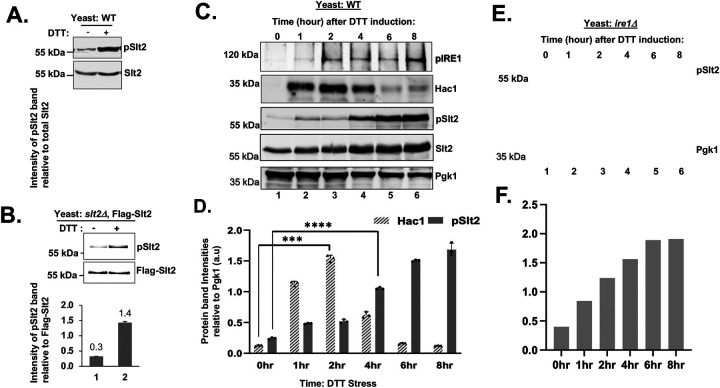
ER-stress activates Slt2 kinase without requiring IRE1. (A) WCEs were subjected to Western blot analysis to detect total Slt2 using a polyclonal antibody and its phosphorylated form (pSlt2) using an antibody specifically designed to detect the phosphorylated residues T202 and Y204 of human ERK1. (Lower panel) The bar diagram depicts the relative intensities of pSlt2 protein bands. (B) WCEs were prepared from the *slt2Δ* strain expressing the Flag-Slt2 from a *GAL1* promoter and subjected to Western blot analysis to detect total Slt2 and pSlt2. (C) WCEs were prepared from WT cells treated with DTT with the indicated time and subjected to Western blot analysis to detect pIRE1, Hac1, pslt2, Slt2 and Pgk1. (D) The intensities of Hac1 and pSlt2 protein bands in the panel (C) were quantified using the ImageJ software and shown in a bar diagram. (E) WCEs were prepared from the *ire1Δ* strain and subjected to Western blot analysis to detect pslt2, Slt2 and Pgk1 proteins. (F) The bar diagram depicts the relative intensities of pSlt2 protein bands shown in the panel (E).

MAPKs are typically activated by phosphorylation at the conserved threonine (Thr or T) and tyrosine (Tyr or Y) residues of the TxY motif of the activation loop (x represents any other amino acid) [1, 2]. Like a typical MAPK, Slt2 contains a conserved TxY motif within the activation loop (T190 and Y192, Fig. S5). Thus, we monitored the phosphorylation status of residues T190 and Y192 by Western blot using an antibody specifically designed to detect the phosphorylated residues T202 and Y204 within the ERK1 activation loop (Fig. S5). The activation loop phosphorylation of Slt2 (pSlt2) was enhanced ~2.5-fold when WT cells were grown in the presence of DTT (Fig. 2A). We also found that the activation loop phosphorylation of Flag-Slt2 was increased when treated with tunicamycin (Fig. 2B). These results are consistent with the earlier studies that MAPK Slt2 is activated in response to ER stress [35].

Next, we investigated the pattern of Slt2 activation during ER stress. WT cells were grown with DTT for 1, 2, 4, 6 or 8 hours. WCEs were prepared and subjected to Western blot analysis using polyclonal antibodies specific for Hac1, Slt2 and Pgk1, alongside phospho-specific antibodies targeting the phosphorylated activation loop of Slt2 and the activation loop of IRE1 (which detects phosphorylated residues S841 and T844) (Fig. S6). As expected, the phosphorylated form of IRE1 (pIre1) was increased within 1 hour of DTT treatment, coinciding with the increase in Hac1 protein level (Figs. 2C and 2D, upper two panels). Notably, the Hac1 protein expression was reduced at the 6 and 8 hours after post-DTT treatment. Interestingly, a modest increase in phosphorylated Slt2 (pSlt2) was observed at 1 and 2 hours of post-DTT treatment (Fig. 2C, middle panel), whereas a pronounced 19-fold increase in pSlt2 levels was observed from the 4^th^ hour onward (Figs. 2C and 2D, middle panel). These results suggest that ER stress response occurs in two distinct phases: an initial phase followed by an adaptive phase.

The initial phase, which is likely a rapid survival response, is primarily mediated by the activated IRE1, leading to the production of Hac1 protein. The second phase involves the activation of both Slt2 and IRE1. During this phase, the IRE1 pathway is partially attenuated, resulting in reduced Hac1 protein production (Fig 2C), while the Slt2 MAP kinase contributes to the restoration of cellular homeostasis and adaptation to ongoing ER stress. These observations help explain the severe sensitivity of *slt2Δ* strain to tunicamycin when grown on a solid medium (Fig. 1B). Together, our results suggest that Slt2 kinase achieves its maximum activation during the adaptive phase of UPR. Moreover, the pattern of pSlt2 level in *ire1Δ* strain closely resembled that of WT cells (Fig. 2E), suggesting that Slt2 activation occurs independently. Interestingly, we also observed that the Slt2 protein levels were increased upon DTT treatments (Fig. 2E), implying that Slt2 transcription may be upregulated during ER stress.

### 3. Rlm1 plays a regulatory role in *HAC1* mRNA splicing.

Two possible mechanisms by which Slt2 likely contributes to ER stress response: (I) It may phosphorylate a substrate that facilitates adaptation to ER stress. (II) Alternatively, it may function through direct interactions with its binding partner(s) or those associated with its pseudokinase counterpart Kdx1(Fig. S7). Potential substrates include several reported proteins, such as transcription regulators Rlm1 [38], Swi4 [39], Sir3 [40] or Rcn2 [41]); protein modifiers Ssb2 [42] or Sod1 [43]); translational repressor Caf20 [44]; signaling regulators Bcy1 [45], Cyclin C [46], or Gga1 [41] (Fig. S8). Potential interaction partners include transcriptional factors (e.g., Crz1, Ccr4, Gat2, Msn2, and Dig1) and signaling components (e.g., Bck1, Mkk1 and Tor1) and protein modifiers (e.g., Sah1 and Mpt5) (Figs. S8 and S9, Saccharomyces Genome Database). Thus, we hypothesized that deletion of an Slt2 substrate or interacting partner involved in ER stress response would result in increased sensitivity to tunicamycin.

**Figure 3:**
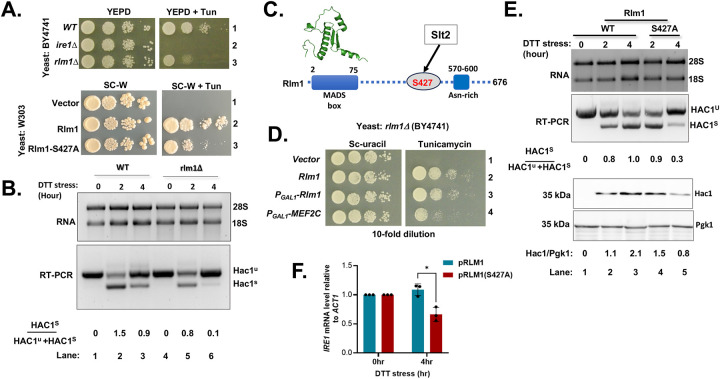
*HAC1* mRNA splicing is reduced in the *rlm1Δ* strain. (A) (Upper panel) WT and *rlm1Δ* yeast strains in BY4741 background were grown on the YEPD medium and the same medium containing tunicamycin (2.5 μg/ml). (Lower panel) A *rlm1Δ* strain (W303 background) expressing a vector plasmid or the same vector expressing Rlm1 or its derivative Rlm1-427A was tested for growth on SC-tryptophan (SC-W) or the same medium containing tunicamycin. (B) WT and *rlm1Δ* strains were grown in the presence of DTT for the indicated time. Total RNA was purified from the DTT-stressed cell (upper panel) and subjected to RT-PCR analysis to monitor the spliced (HAC1^s^) and un-spliced (HAC1^u^) *HAC1* mRNAs (middle panel). The intensities of *HAC1* mRNAs were quantified using the ImageJ software and shown. (C) The schematic representation of Rlm1 protein containing a MADS-box and an Asn-rich domain. The numbers indicated the amino acid numbers. The predicted Alpha-Fold structure of the MAD-box domain is shown. (D) The *rlm1Δ* strains in the BY4741 background expressing a vector plasmid or the same vector expressing Rlm1 or human MEF2C from its own promoter (row 2) or from a *GAL1*-promoter were tested for growth on SC-uracil and the same medium containing tunicamycin (2.5 μg/ml). (E) (Upper panel) Total RNA and WCEs were purified from the DTT-stressed *rlm1Δ* strains expressing WT or S427A mutant of Rlm1. Total RNA was subjected to RT-PCR analysis to monitor the spliced (HAC1^s^) and un-spliced (HAC1^u^) *HAC1* mRNAs. (Lower panel) WCEs were subjected to Western blot analysis to detect Hac1 and Pgk1 proteins. The relative intensities of Hac1 protein bands are shown. (F) Expression of *IRE1* mRNA levels analyzed by RT-qPCR in the *rlm1Δ* strain expressing the WT and the S427A mutant of Rlm1 treated with DTT for 4h.

The *rlm1Δ*, *sod1Δ* and *rcn2Δ* strains were sensitive to tunicamycin (Fig. 3A and Fig. S8). *RCN2* encodes a protein of unknown function, *SOD1* encodes a superoxide dismutase, and *RLM1* encodes a transcription factor. In both *rcn2Δ* and *sod1Δ* strains, *HAC1* mRNA splicing levels were comparable to those observed in WT strain (Fig. S8C). In contrast, a reduced *HAC1* mRNA splicing (Fig. 3B and Fig. S8C) in the *rlm1Δ* strain suggested that Rlm1 plays a direct regulatory role in this splicing process. To further validate this observation, we disrupted the *RLM1* gene in the W303 background, another widely used laboratory yeast strain. The *rlm1Δ* strain in the W303 background was also sensitive to tunicamycin (Fig. 3A, lower panel and Fig. S10), in which splicing of *HAC1* mRNA was notably reduced when grown in the presence of DTT for 4 hours (Fig. S10B), supporting further that Rlm1 plays a regulatory role in *HAC1* mRNA splicing.

Yeast cells are also known to express an Rlm1 paralog, Smp1. Unlike *rlm1Δ* strain, the *smp1Δ* strain displayed normal growth on the tunicamycin medium (Fig. S10), suggesting that this paralog likely functions independently. However, we generated a *rlm1Δsmp1Δ* double deletion strain, which, like *rlm1Δ* strain, was also sensitive to tunicamycin (Fig. S10). Consistently, in the *rlm1Δsmp1Δ* strain, both *HAC1* mRNA splicing and Hac1 protein production were markedly reduced following a 4-hour DTT treatment (Fig. S10), supporting a role for Rlm1 in facilitating IRE1-mediated *HAC1* mRNA splicing.

Rlm1 contains an N-terminal MADS-box domain and a largely unstructured C-terminal domain (Fig. 3C). The MADS-box specifically binds to a consensus CTA[T/A]4TAG motif within the target promoters, while the C-terminal domain facilitates assembly of general transcription factors, thus activating transcriptions of numerous genes involved in cell wall stress response[38]. Phosphorylation at Ser427 of the C-terminal domain by Slt2 is known important for Rlm1 function [47] (Fig. 3C). Notably, mutation at S427 of Rlm1 impaired growth on the tunicamycin medium (Fig 3A, lower panel, row 3), indicating that phosphorylation of at this site is crucial for an effective ER stress response.

A BLAST homology search identified that MEF2 (Myocyte enhancer factor 2) family of transcription factors (e.g., MEF2A, MEF2B, MEF2C and MEF2D) are the closest human orthologs of Rlm1. The highest sequence conservation is found within the N-terminal region (residues 1–80), which constitutes the MADS-box domain. (Fig. S11A). Among these, MEF2A and MEF2C showed highest homology, each showing 46.32% identity and low E-values (Figs. S11B and S11C). MEF2C was selected for further analysis. Notably, the Rlm1’s phosphorylation site Ser427 aligns with the Ser361 of MEF2C (Fig. S11D). Expression of MEF2C from a GAL1 promoter complemented the function of Rlm1 (Fig. 3D, row 4 and Fig. S12), consistent with the bioinformatic prediction that MEF2C is a functional ortholog of Rlm1.

Next, we investigated if *HAC1* mRNA splicing was facilitated by phosphorylation at the residue S427 by Slt2. An Rlm1-S427A mutant was expressed in the *rlm1Δ* strain and monitored *HAC1* mRNA splicing. *HAC1* mRNA splicing was reduced almost 3-fold following 4 hours of DTT treatment in the strain expressing Rlm1-S427A mutant compared to strain expressing a WT allele (Fig. 3E, RT-PCR). Consistently, Hac1 protein expression was also lower in the *rlm1Δ* strain expressing a Rlm1-S427A mutant (Fig. 3E, lower panels, lane 5). Together, these results suggest that Rlm1 promotes *HAC1* mRNA splicing in a manner dependent on its phosphorylation at S427 of Rlm1 by Slt2.

As *HAC1* mRNA splicing depends on the abundance of *IRE1* mRNA or protein, we investigated the level of *IRE1* mRNA in the *rlm1Δ* strains expressing either the WT allele or the S427A mutant of Rlm1. Under normal growth condition, both strains showed comparable levels of *IRE1* mRNA (Fig 3F). However, upon induction of ER stress for 4 hours, the levels of *IRE1* mRNA were decreased in the strain expressing the S427A mutant (Fig 3F). These results further support the notion that phosphorylation of Rlm1 at S427 by Slt2 is important for proper *IRE1* expression.

### 4. Overexpression of IRE1 rescues UPR activity in the *rlm1Δ* strain.

**Figure 4:**
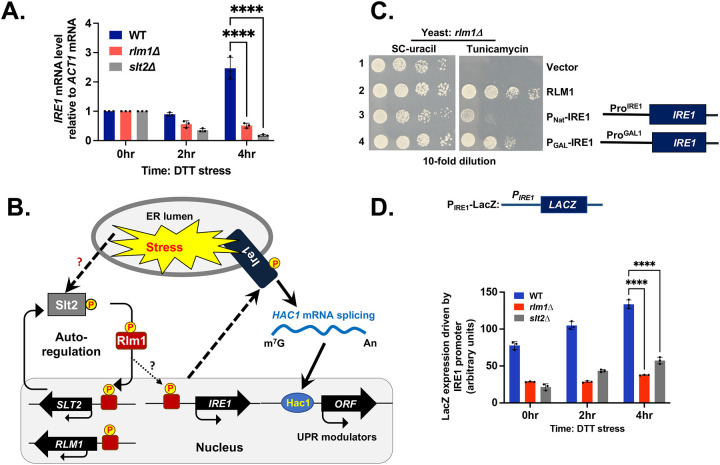
Over-expression of IRE1 rescues UPR in the *rlm1Δ* strain. (A) Expression of *IRE1* mRNA in WT, *rlm1Δ* and *slt2Δ* strains during an ER stress shown (****p-value<0.0001, paired t-test). (B) During ER stress, IRE1, Slt2 and Rlm1 are activated by phosphorylation, as denoted by the encircled “P” symbol. The phosphorylated for of Rlm1 promotes the transcription of SLT2, RLM1 and IRE1 genes, as indicated by black arrows. (C) The *rlm1Δ* strain expressing Rlm1 or IRE1 from the indicated promoter were tested for growth on SC-uracil and the same medium containing tunicamycin. (D) Expression of LacZ driven by *IRE1* promoter in WT, *rlm1Δ* and *slt2Δ* strains shown in a bar diagram (****p-value<0.0001, paired t-test).

Based on our new findings and previously published data [48], we hypothesize that Rlm1 may bind to the *IRE1* promoter and enhance its transcription during ER stress. The new findings include: (I) the *rlm1Δ* strain containing a plasmid harboring the IRE1 open reading frame along with adjacent genomic regions (selected from a yeast tiling library) was resistant to tunicamycin (Fig. S13). (II) In both *slt2Δ* and *rlm1Δ* strains, the *IRE1* mRNA levels were significantly reduced following a 4-hour DTT treatment (Fig. 4A). Previous studies showed that Rlm1 bind to its own promoter as well as the *SLT2* promoter, thereby autoregulating transcription of both genes[48] (Fig. 4B) and the mRNA levels of both *SLT2* and *IRE1* increased during ER stress [49, 50].

To evaluate the above hypothesis, we sought to bypass the endogenous regulation of Ire1 by over-expressing *IRE1* from a constitutive *GAL1* promoter in both *slt2Δ* and *rlm1Δ* strains. For this purpose, two high-copy-number plasmids were generated to express *IRE1*: one from its native promoter (i.e., *P*_*Nat*_-*IRE1*) and the other from the constitutive *GAL1* promoter (i.e., *P*_*GAL1*_-*IRE1*). The *slt2Δ* or *rlm1Δ* strain carrying P_Nat_-IRE1 failed to grow on the tunicamycin medium (Fig. 4C, row 3), whereas the same strain carrying *P*_*GAL1*_-*IRE1* grew (Fig. 4C, row 4). These results suggested that *GAL1*-driven *IRE1* bypassed the requirement of the Rlm1-dependent transcriptional activation of *IRE1*, further supporting the role of Rlm1 in regulating *IRE1* expression.

To further investigate our findings, we generated a P_IRE1_-LacZ reporter construct by fusing the *IRE1* promoter region (1000 base pairs upstream of the ORF, Fig. S14) to the *LacZ* gene. The LacZ expression was monitored as we described previously [51]. In WT cells, LacZ expression was increased following DTT treatment for 2 and 4 hours (Fig. 4D). However, in both *slt2Δ* and *rlm1Δ* strains, the LacZ expression was markedly reduced (Fig. 4D), particularly at 4 hours of DTT treatment. These findings further confirm that Rlm1 may function as a transcription activator of *IRE1*.

### 5. The promoter of *IRE1* contains an Rlm1-specific binding site (Rlm1-BS).

**Figure 5:**
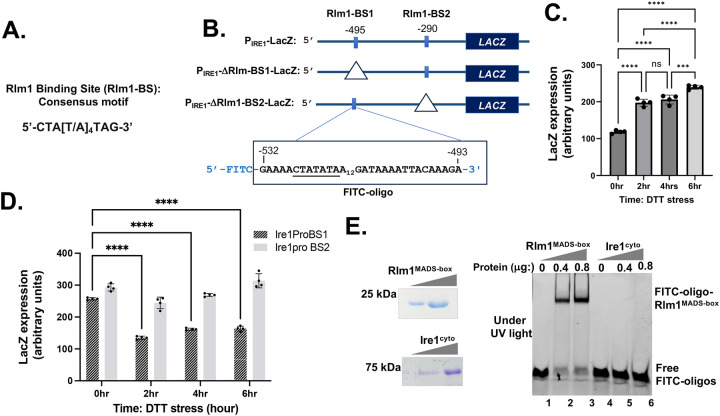
The *IRE1* promoter contains an Rlm1 binding site (Rlm1-BS). (A) The consensus nucleotide sequence motif of the Rlm1 binding site. (B) The schematic representation of *IRE1* promoter driven LacZ constructs. Two potential Rlm1 binding sites (Rlm1-BS1 and RLm1-BS2) are shown in a solid rectangle. The triangle signs indicate that Rlm1-BS1 or Rlm1-BS2 are removed. The nucleotide compositions of the Rlm1-BS2 site are shown. (C) Expression of LacZ from the P_IRE1_-LacZ construct in WT cells after DTT treatments (***p-value<0.001, ****p-value<0.0001, paired t-test, ns = non-significant). (D) Expression of LacZ from the P_IRE1_-LacZ construct and its derivates are shown in a bar diagram (****p-value<0.0001, paired t-test). (E) (Left panels) Purified Rlm1^MADs-box^ and Ire1^cyto^ proteins were resolved by an SDS-PAGE and stained with Coomassie blue. (Right panel) A fluorescein isothiocyanate (FITC)-tagged DNA oligonucleotide (FITC-oligo) corresponding to the Rlm1-BS1 target site as shown in (B) was annealed with its complementary strand and mixed with the purified Rlm1^MADs-box^ or Ire1^cyto^ in a binding buffer. The reaction mixtures were then resolved on a native gel and visualized under UV light.

The JASPAR database (https://jaspar.elixir.no) predicts two Rlm1-specific binding sites (Rlm1-BS) with ~80% sequence homology upstream of the *IRE1* open reading frame: one located at position −495 (BS1) and the other at −290 (BS2) relative to the AUG start codon, which is designated as +1 (Fig. 5A). To test the functional significance of Rlm1-BSs in the *IRE1* promoter, we engineered the P_IRE1_-LacZ reporter construct to delete the potential Rlm1-BSs at positions −495 and −290, thus generating P_IRE1_-ΔRlm1-BS1-LacZ and P_IRE1_-ΔRlm1-BS2-LacZ constructs, respectively (Fig. 5B). In wild type cells, the lacZ expression from the P_IRE1_-LacZ or P_IRE1_-ΔRlm1-BS2-LacZ constructs was increased following DTT treatment for 2, 4 and 6 hours (Fig. 5C), whereas a significant decrease in LacZ expression was observed from the P_IRE1_-ΔRlm1-BS1-LacZ construct (Fig. 5D). These results suggest that the Rlm1 binding site at position −495 is functionally important for IRE1 promoter activity.

To confirm our findings, we performed an in vitro binding assay with the purified Rlm1 protein residues 1– 75 (Rlm1^MADS-box^) and the Rlm1 binding site located at the position −495 of *IRE1* promoter (Fig. 5B). Two DNA oligonucleotides were synthesized from a commercial vendor (SIGMA, USA): one was a fluorescein isothiocyanate (FITC)-tagged DNA oligonucleotide (FITC-oligo) corresponding to the target site and other was its complementary strand (Fig. 5B). Both oligonucleotides were annealed and incubated with the purified Rlm1^MADS-box^ protein (Fig. 5E, right panel) in a binding buffer. As a control, the same double stranded DNA oligo was also incubated with the purified cytoplasmic domain of IRE1 protein residues 658–1115 (Ire1^cyto^) (Fig. 5E, left panel). The reaction mixtures were then resolved on a native gel and visualized under the UV light. A high-molecular-weight protein band was observed when FITC-oligo was incubated with Rlm1^MADS-box^ protein but not with Ire1^cyto^ (Fig. 5E), indicating that the FITC-oligo specifically binds to the Rlm1^MADS-box^ protein. Together, our results show that the promoter of *IRE1* contains an Rlm1 binding site.

### 6. Human MAP kinases ERK1, ERK2 and ERK5 complement the Slt2 function.

**Figure 6:**
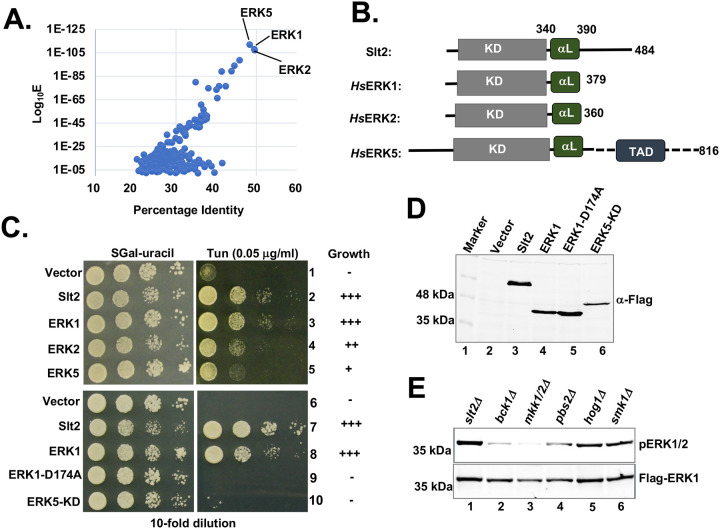
Human ERK1, ERK2 and ERK5 complement yeast Slt2. (A) The Slt2 protein sequence was used in a BLAST search against the human genome database. The percentage identify and corresponding Log10E values are plotted. (B) The schematic representation of Slt2, human ERK1 (hsERK1), ERK2 (hsERK2) and ERK5 (hsERK5) protein kinases. The kinase domain (KD), helix αL and the transcription activation domain (TAD) are indicated. (C) The *slt2Δ* strains expressing the indicated Flag-tagged MAP kinase, or their derivatives were grown on SGal-uracil medium or the same medium containing tunicamycin. The relative growths are indicated by the sign “+”. (D) WCEs were prepared from *slt2Δ* strains expressing the indicated Flag-tagged MAP kinase and subjected to Western blot analysis using anti-flag antibody. (E) WCEs were prepared from the indicated yeast strains expressing the Flag-tagged ERK1 and subjected to Western blot analysis to monitor pERK1 and total ERK1.

The NCBI BLAST search against the human genome database showed that ERK1, ERK2 and ERK5 kinases were close orthologs of Slt2 kinase with sequence identities 48.32% (E value = 8×10^−113^), 49.43% (E value = 3×10^−109^) and 49.57% (E value = 2×10^−108^), respectively (Fig. 6A). The multiple protein sequence alignment showed that the kinase domains (KDs) of Slt2 (residues 23–318), ERK1 (residues 42–330), ERK2 (residues 25–313) and ERK5 (residues 54–346) exhibited the highest degree of homology (Fig. 6B). Notably, at the C-terminal end of the kinase domains, all four kinases share a common sequence element with 50% identity. This element adopts a helical structure, known as “helix-αL” (Fig. 6B and Fig. S15), as seen in the crystal structures of ERK1, ERK2 and ERK5 (Fig. S15). To assess the functional ortholog of Slt2, we tested whether ERK1, ERK2 and ERK5 could compensate the loss of Slt2 kinase in the context of ER stress response. The *slt2Δ* stain was transformed with a plasmid expressing ERK1, ERK2 or ERK5 from a galactose inducible *GAL1* promoter. Transformants were then grown on the medium containing galactose (2%) with or without tunicamycin.

The *slt2Δ* stain expressing the ERK1, ERK2 or ERK5 grew on the medium containing tunicamycin (Fig. 6C). Notably, we observed that cells expressing the ERK1 grew better than cells expressing ERK2 or ERK5. In contrast, the same cells expressing a kinase inactive ERK1-D174A mutant (D = Aspartate of the catalytic HRD motif) or EKR5-KD failed to grow on the medium containing tunicamycin (Fig. 6C, rows 9 and 10). Western blot shows the expression of both ERK1-D174A and ERK5-KD proteins (Fig. 6D, lanes 5 and 6), suggesting that both ERK1-D174A and ERK5-KD were unable to complement the loss of Slt2. These findings suggest that kinase activity of ERK1 was important to complement Slt2 function under an ER stress condition. These results suggest that ERK1, ERK2 and ERK5 are functional orthologs of Slt2 in the context of ER stress response.

The structural analysis of ERK1, ERK2 and ERK5 showed that helix-αL interacts with their kinase domain (Fig. 6B and Fig. S15), indicating that it might regulate the kinase domain function. In the predicted Slt2 structure, the helix-αL adopts an elongated conformation while bound to its kinase domain (Fig. S15). To assess the importance of the elongated helix-αL within Slt2 kinase, we systematically removed the C-terminal residues. Subsequently, we expressed these truncated proteins (comprising of residues 1–440, 1–400 and 1–355) in the *slt2Δ* strain using a *GAL1* promoter inducible by galactose. The truncated Slt2 proteins (Slt2^1−440^ and Slt2^1−400^) consisting of the kinase domain and the helix-αL fully complemented the Slt2 function (Fig. S16). However, the truncated Slt2 protein consisting of the kinase domain and a shorter helix-αL, mutant Slt2^1−355^) failed to restore the Slt2 function (Fig. S15). Western blot showed that the expression of truncated protein consisting of residues 1 to 355 (Slt2^1−355^) was similar to the WT Slt2 protein (Fig. S16). These results indicate that the C-terminal end of Slt2 protein has a redundant function. Additionally, these results suggest that the sole Slt2 kinase domain is non-functional, while the helix-αL plays an important role in its kinase domain function.

To further investigate the role of kinases Bck1 or Mkk1/2 in the activation of EKR1, we examined the phosphorylation status of ERK1 within its activation loop in the *bck1Δ mkk1/2Δ* strain. We observed that the phosphorylation of ERK1 was substantially reduced in both *bck1Δ* and *mkk1/2Δ* strains (Fig. 6E, lanes 2, and 3), but not in strain lacking *pbs1Δ*, *hog1Δ* and *smk1Δ* strains (Fig. 6E, lanes 4, 5, and 6). These results suggest that Bck1 and Mkk1/2 activate ERK1, akin to their role in activation of the kinase Slt2.

### 7. ER stress activates MAP kinase ERK1 in human cells.

**Figure 7:**
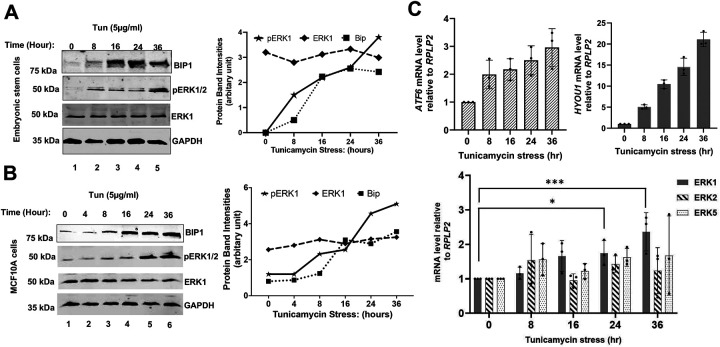
ERK1/2 phosphorylation increases in response to ER stress. (A) (Left panel) WCEs were prepared from embryonic stem cells treated with tunicamycin and subjected to Western blot analysis to detect BIP1, pERK1/2 and ERK1/2. (Right panel) The protein band intensities are shown in a line diagram. (B) (Left panel) WCEs were prepared from MCF10A non-tumorigenic cells after tunicamycin treatment and subjected to Western blot analysis to detect BIP1, pERK1/2 and ERK1. (Right panel) The protein band intensities are shown in a line diagram. (C) (Upper panel) RT-qPCR was used to measure the relative expression of *ATF6* and *HYOU1* mRNAs in human embryonic stem cells during an ER stress. Experiments were repeated thrice. The average values with standard deviations are shown. (Lower panel) RT-qPCR was used to measure relative expression of ERK1, ERK2 and ERK5 mRNAs in human embryonic stem cells after tunicamycin treatment. The average values of three experiments with standard deviations are shown (***p-value<0.001 and *p-value<0.05, paired t-test).

To investigate the role of ERK kinases in the human ER-stress response, we analyzed the phosphorylation status of ERK1 and ERK2 kinases in human embryonic stem cells treated with tunicamycin. As expected, treatment with tunicamycin led to the increased expression of BIP1 (Fig. 7A), an ER stress marker and a downstream target of the IRE1-XBP1 pathway. Interestingly, we observed a marked increase in the phosphorylated ERK level (pERK1), which peaked at 36 hours following tunicamycin treatment (Fig. 7A). We also monitored the mRNA levels of ER stress sensor ATF6 and one of its targets, HYOU1 as reported earlier[52]. Both ATF6 and HYOU1 mRNA levels were increased when cells were treated with tunicamycin (Fig. 7C, upper panel). To be noted, mRNA levels of *ERK1* was significantly increased in the same cells treated with tunicamycin for 24 and 36 hours (Fig. 7C, lower panel). These results suggest that ER stress induces ERK1 activation in the human embryonic stem cells during the adaptive stage of UPR.

To further confirm our results, we examined ERK1 phosphorylation in the non-tumorigenic MCF10A and primary ovarian surface epithelium cells (OSE) treated with tunicamycin for 36 hours. As expected, BIP1 expression was increased immediately after exposing cells with tunicamycin in both MCF10A (Fig. 7C) and OSE cells (Fig. S17). A basal level of pERK1/2 was observed when cells were treated with tunicamycin in both cell types (Fig. 7C and Fig. S17); however, the pERK1/2 level increased markedly after 24 hours of tunicamycin treatment (Fig. 7C and Fig. S17). Taken together, these findings support the conclusion that ER stress rapidly activates IRE1, while both IRE1 and ERK1 kinases contribute to adaptive stage of UPR, analogous to coordinated activation of both IRE1 and Slt2 in yeast cells (Fig. 2).

## DISCUSSION

Our data provide strong evidence that MAP kinase Slt2 plays a crucial role in UPR by directly influencing the splicing of *HAC1* mRNA (Fig. 1). We also demonstrate that the cellular response to ER stress occurs at least two distinct phases (Fig. 2): An initial phase primarily driven by IRE1 and a subsequent adaptive phase involving both IRE1 and Slt2. During this adaptive phase, the IRE1 pathway is likely attenuated, leading to reduced expression of the Hac1 protein. Concurrently, Slt2-mediated responses come into play to promote adaptation and recovery by activating UPR genes through an alternative route involving its downstream target, Rlm1. Specifically, we show that Rlm1 binds to the IRE1 promoter, and that over-expression of IRE1 compensates for the loss of Rlm1 (Figs. 3, 4, and 5). Furthermore, we show that human ERK1 and the transcription factor MEF2C can functionally substitute for yeast Slt2 and Rlm1, respectively (Figs. 6 and 3). A similar pattern of IRE1 and ERK1 activation is also observed in human primary and stem cells, resembling the activation profile seen in yeast cell (Figs. 2, 6 and 7). Together, these findings reveal a novel crosstalk between the MAPK Slt2/ERK1 and IRE1-mediated UPR signaling pathways.

### The MAPK Slt2 pathway and ER stress-dependent proteostasis

Multiple prior studies highlighted that both IRE1 and MAPK Slt2 contribute to ER proteostasis [35, 53, 54]. The *slt2Δire1Δ* double mutant displays greater sensitivity to ER stress compared to the corresponding single mutants [35], suggesting that the Slt2 pathway initiates an additional signaling pathway in response to ER stress. However, their experiments did not define a critical threshold at which the decline in cellular fitness becomes prominent. The molecular association between Slt2 and IRE1 pathways and their relationship to maintaining ER proteostasis still remain unclear.

Research on the involvement of Slt2 in UPR[35, 54, 55] has primarily focused on the *slt2Δ* cells grown in the presence of ER stressors for a short duration (one hour). For instance, Chen et al. (2005) showed comparable levels of *HAC1* mRNA splicing between WT and *slt2Δ* strains after one hour of growth under an ER stress condition[35]. Similar findings were obtained in our experiments (data not shown). However, these results alone do not explain why the *slt2Δ* strain cannot sustain ER stress and are severely sensitivity to tunicamycin (Fig. 1B). One plausible explanation is that only one of hour of stress is insufficient to see any effect because the activation of pro-survival strategies relies upon the duration of stress signals.

A study conducted by Rousseau and Bertolotti (2016) showed that the expression levels of chaperones for the 26S proteasomal complex (e.g., Adc17, Hsm3, Nas6, Nas2 and Rpn14) were significantly reduced in the *slt2Δ* strain that was grown under a condition of ER stress induced by tunicamycin for 4 hours[36]. Based on these findings, they propose that the tunicamycin sensitivity of the *slt2Δ* strain is likely due to defects in assembly of the functional 26S proteasome complex that clears the unfolded proteins from cells during ER stress. However, yeast strains lacking either of these chaperones form normal colonies on the tunicamycin medium (Fig. S18), underscoring the importance of Adc17, Hsm3, Nas6, Nas2 or Rpn14 in ER stress response. So, the precise role of Slt2 in ER stress remains to be conclusively determined.

Compared to the isogenic WT cells, we observed a significant reduction (>50%) in both splicing of *HAC1* mRNA and Hac1 protein production when ER stress was prolonged (Figs. 1 and 2), This reduced splicing may be associated with a decrease in the functionality of the IRE1-RNase and/or tRNA ligase functions, while the decrease in the Hac1 protein production is associated with the reduced translation efficiency of the spliced *HAC1* mRNA. Although precise role of Slt2 in *HAC1* mRNA translation remains under investigation, our data strongly support a model that Slt2 functions in a signaling pathway that directly connects the IRE1-Hac1 mediated UPR.

**Figure 8:**
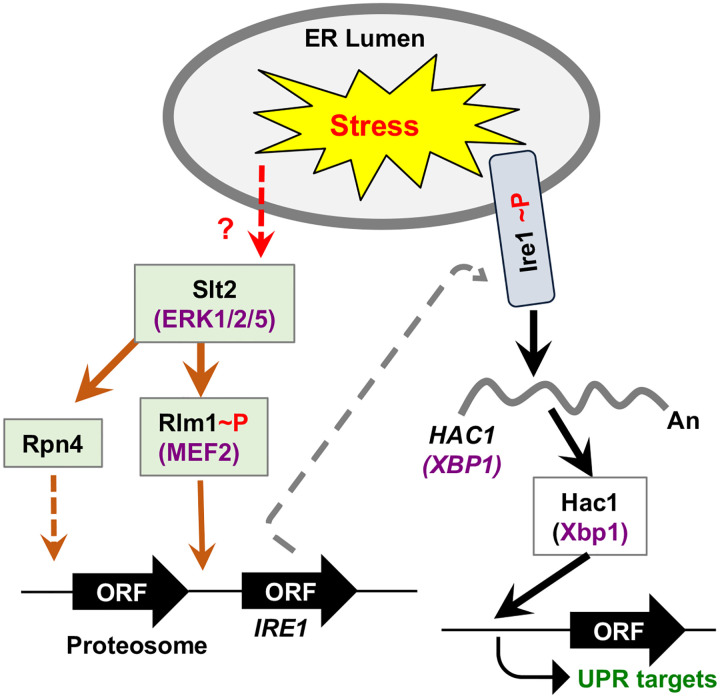
Proposed link between IRE1 and MAPK Slt2 signaling pathways. The ER stress activates both IRE1 (light blue box) and MAP kinase Slt2 or ERK1 (colored in brick red). Active IRE1 removes the intronic sequence (orange wavy line) from the *HAC1* or *XBP1* mRNA (green wavy line). The matured *HAC1/XBP1* mRNA translates Hac1/Xbp1 protein that binds to the upstream sequence of the UPR genes, thus activating their transcription. Active Slt2 promotes expression of chaperones of proteosome via Rpn4 as reported earlier [36] as well as phosphorylates and activates the transcriptional activator Rlm1 or human counterpart MEF2C. Rlm1 induces the transcription of *IRE1*, thus facilitating *HAC1* mRNA splicing.

Constitutive Hac1 expression in the *slt2Δ* strain failed to restore ER stress tolerance (Fig. 1), indicating that the IRE1-mediated UPR relies on additional signals beyond Hac1 alone. These additional signals are presumed to generate specific transcriptional and/or translational programs appropriate for the specific physiological requirements. One such transcriptional program may originate from the MAPK Slt2 pathway, via phosphorylation of its downstream target Rlm1. Notably, yeast cells lacking Rlm1, a substrate of Slt2, exhibit impaired ER response, which can be partially rescued by constitutive expression of IRE1(Fig. 4). This finding suggests that Rlm1 may play a role in *IRE1* expression, thus modulating transcriptional output of the UPR genes for sustained stress adaptation. Supporting this model, we provide strong evidence that the MAD-box domain of Rlm1 binds directly to the *IRE1* promoter (Fig. 5), and that LacZ expression driven by *IRE1* promoter is significantly upregulated during ER stress condition (Fig. 5). Collectively, our results uncouple a new molecular link between the MAPK Slt2/ERK1 pathway and IRE1-driven UPR through Rlm1 (Fig. 8).

### The Helix-αL in MAP kinase function

The helix-αC is a dynamic regulatory element within the protein kinase domain[56]. In the active state of a kinase domain, the helix-αC’s N-terminal end engages with the phosphorylated activation loop. The distance between the N-terminal end of the helix-αC and the phosphorylated activation loop defines the open and closed conformations of the kinase domain which is crucial for the kinase catalysis[56]. The helix-αC of ERK1/2 kinase domain interacts with the helix-αL located at the adjoining C-terminal end[57]. In this study, we show that the analogous helix-αL of the Slt2 kinase domain is important for its role in ER stress response (Fig. 6B and Fig. 6C). These findings indicate that the helix-αC•helix-αL interaction likely controls the overall conformation and activation of both kinases ERK1 and ERK2. However, the direct evidence supporting this functional interaction and the role of the extended helix-αL in the Slt2 kinase domain activation remains to be determined.

### The MAPK substrates in human UPR

The MAP kinase Slt2 displays its closest sequence similarity with members of the mammalian MAPK family, including ERK1, ERK2 and ERK5 (Fig. 6). It is worth mentioning that none of these mammalian MAP kinases displayed more than 50% identity to Slt2. Nonetheless, ERK1, ERK2 and ERK5 could compensate the loss of Slt2 in the context of ER stress response (Fig. 6). To be noted that ERK5, but not ERK1 or ERK2 could compensate for the loss of Slt2 kinase in the context of genotoxic stresses [19] (Fig. S19). Furthermore, we observe that fission yeast Spk1, an ortholog of Slt2, did not have any compensatory effect regarding the cellular response to ER stress (Fig. S19). These observations collectively suggest that significant differences exist in the regulation of MAPK pathways across different species, despite the evolutionarily conservation of these stress-related pathways.

Both ERK1 and ERK2 appear to be constitutively active in HEK293 human cells (Fig. S20). This could be attributed the fact that active ERK1/2 are known to phosphorylate a diverse group of substrates (e.g., protein kinases, phosphatases, and regulators of apoptosis) to control multiple signaling pathways[58–61]. Current evidence suggests that ERK1/2 specificity is influenced by their subcellular location (e.g., nucleus vs cytoplasm), cellular context (e.g., stress response), and the presence of substrate docking site (e.g., DEF motif[62]). However, the precise molecular mechanisms governing the broad substrate specificity remain unknown. While our findings suggest that MEF2C could be a potential target of ERK1, the key challenge remains in identifying a substrate that is specifically involved in UPR.

### Experimental procedures

#### Yeast strains, growth, and gene disruption

Yeast *S. cerevisiae* strains were grown in the standard YEPD medium (1% yeast extract, 2% peptone and 2% dextrose) or defined synthetic complete (SC) medium (0.17% yeast nitrogen base, 0.5% ammonium sulphate, 2% glucose and all amino acids). The genomic DNA of the *ire1::HphMX* or *rlm1::KanMX* strain was used as a template to amplify the *HphMX* or *KanMX* cassette using primers annealing ~200-bases upstream and downstream of the *IRE1 or RLM1* open reading frame. The amplified PCR product was used to disrupt the *IRE1* and *RLM1* genes. The list of yeast strains used in this study is shown in Table 1.

#### Preparation of plasmids:

Plasmids were generated using the standard gene manipulation techniques. Mutation was generated by fusion PCR using standard protocols. The desired mutation in each plasmid was confirmed by Sanger sequencing. The list of plasmids used in this study is shown in Table 2.

#### Whole cell extract preparation from yeast and Western blot analysis

Yeast cells were grown in YEPD or Synthetic complete (SC) medium without appropriate nutrients until the OD_600_ value reached ~0.5 to 0.6. DTT (5 mM) or tunicamycin (0.5 μg/ml) was added to the medium to induce ER stress, and cells were harvested after 2 hours (unless otherwise indicated). Whole cell extracts (WCEs) were prepared by TCA method as described previously [51]. Proteins were then fractioned by SDS–PAGE and Western blot analysis was performed using appropriate antibody (See Table 3). All experiments were repeated at least two times.

#### RNA analysis, reverse transcriptase (RT)-PCR, and real time (RT)-quantitative PCR (RT-qPCR)

Yeast cells were grown in YEPD or SC medium without appropriate nutrients at 30°C until they reached an OD_600_ value between 0.5 and 0.6. The ER stressor DTT (5 mM) or tunicamycin (1 μg/ml) was added to the medium and cells were grown further for another 2 hours (unless otherwise indicated). Human cells were grown as described below. Total RNA was isolated using the RNeasy mini kit (Qiagen). Purified RNA was quantified using a Nanadrop spectrophotometer (ND-1000, Thermo Scientific). For *HAC1* mRNA, purified RNA was used to synthesize the first strand cDNA by a Superscript^™^-III reverse transcriptase (Invitrogen 18080–093) and a reverse primer (5ʹ-CCCACCAACAGCGATAATAACGAG-3ʹ) that corresponded to nucleotides +1002 to 1025. To assay *HAC1* mRNA splicing, the synthetic cDNA was then PCR-amplified using a forward primer (5ʹ-CGCAATCGAACTTGGCTATCCCTA CC-3ʹ) that corresponded to nucleotides +35 to 60 and a reverse primer (5ʹ-CCCACCA ACAGCGATAATAACGAG-3ʹ) that corresponded to nucleotides +1002 to 1025. The PCR-amplified products were then run on a 1.5% agarose gel to separate spliced (HAC1^s^) and un-spliced (HAC1^u^) forms of *HAC1* mRNA. Quantities of HAC1^s^ and HAC1^u^ were measured using ImageJ. Experiments were repeated at least two times.

For qPCR, appropriate cDNA was synthesized using random primers (NEB, USA) from total RNA. The concentration of each cDNA sample was quantified using the Nanadrop spectrophotometer. For each qPCR reaction, 4–5 ng of cDNA was used as template to quantify the expression levels of specific mRNAs. The qPCR was performed using gene-specific primers (Table 4) and SYBR^®^ Green PCR Master Mix (Applied Biosystems, Cat. No. A25742) on an Applied Biosystems StepOne^™^ real-time PCR system. All reactions were conducted in triplicate. Relative mRNA levels were quantified using the ΔΔCt method, with normalization against *ACT1* (for yeast samples) and *RPLP2* (for human samples) as internal reference genes.

To make sure all our primers worked efficiently and gave reliable results, we created a standard curve for each target gene. Each target gene was PCR-amplified from the cDNA using the specific primers, purified the PCR products and then prepared a series of dilutions ranging from 100 to 1 billion copies for each PCR product. We ran qPCR on these dilutions and recorded the Ct (cycle threshold) values using the StepOne^™^ Software v2.2.2 (Applied Biosystems). From the data, we calculated how efficient each primer set was, using the formula: E = 10^(−1/slope), where the slope comes from the standard curve. All primer pairs used in this study demonstrated comparable amplification efficiencies, thereby justifying the use of the ΔΔCt method for relative quantification in subsequent qPCR experiments.

#### Protein purification and Electrophoretic Mobility Shift Assay (EMSA)

For protein expression in bacteria (Rlm1^MADS-box^ and Ire1^cyto^) we used BL21(DE3) cells. Recombinant proteins were purified by nickel agarose as described earlier [63]. Purified proteins were mixed with FITC-tagged DNA oligo (Sigma-Aldrich) in a binding buffer (13 mM Tris-HCl pH 7.0, 1.5 mM MgCl2). Reaction mixtures were incubated at 30°C for 45 minutes with intermittent tapping. The reaction mixtures were separated using a 12% TBE (Tris-borate-EDTA) gel, and RNA was visualized under UV light.

#### Human cell culturing and whole cell extract (WCE) preparation

Human embryonic stem cells (H9; WA09, Wicell Research Institute, Madison, Wisconsin, USA, licensed to Prof. Nadege N Gouignard^1^) with a normal karyotype were used as wildtype hESCs line. Cells were grown and expanded on growth-factor reduced Geltrex-coated dishes (Gibco, #A1413302) in mTeSR^™^1 medium (Stem Cell Technologies, #85850) until they reached ~80% confluence. The medium was replaced daily, cells were split every 5–7 days with accutase (Stem Cell Technologies, #07920)33 and plated at a 1:6 dilution onto the Geltrex-coated plates.

Non-tumorigenic MCF10A cells were grown in DMEM/F12 media (Sigma-Aldrich) containing 5%horse serum, 20 ng/mL hEGF (Peperotech), 0.5 ug/ml Hydrocortisone (Sigma-Aldrich), 100 ng/ml Cholera Toxin (Sigma-Aldrich), 10 ug/mL Insulin (Sigma-Aldrich) and Pen/Strep (Thermo Scientific). Ovarian surface epithelial (OSE) cells were isolated as described earlier[64]. OSE was maintained in cell culture medium consisting of 1:1 Medium 199 and MCDB105 medium (Sigma-Aldrich) with 10% FBS, 10 ug/mL Insulin Solution (Sigma-Aldrich), 10 ng/mL hEGF (Peperotech) and Pen/Strep (Thermo Scientific)[65]. Cells were routinely tested and deemed free of PlasmotestTM Mycoplasma Detection Kit (InvivoGen, San Diego, CA). Authenticity of the cell lines used were confirmed by STR characterization at IDEXX Bioanalytics Services (Columbia, MO).

Normal human ovarian epithelial tissues were collected from individuals with the tumor-free ovary, following the Institutional Review Board approved protocol at Medical College of Wisconsin. Ovarian surface epithelial cells (OSE) were cultured by scraping the surface epithelium of normal ovarian tissues. OSE cells were cultured in MCDB105/M199 medium (Sigma-Aldrich, St. Louis, MO) (1:1 mixture) supplemented with 10% FBS, human epidermal growth factor and insulin in the presence of tunicamycin.

HEK293FT (Invitrogen R70007) cells were cultured in a high glucose Dulbecco’s modified Eagle’s medium (DMEM) (Thermo Scientific) medium supplemented with 10% fetal bovine serum (Thermo Scientific), 100 U of penicillin G (Thermo Scientific), 100 μg/ml streptomycin (Thermo Scientific), and 6 mM L-glutamine (Thermo Scientific). Cells were maintained at 37°C with 5% CO_2_. Cells were seeded in a 6-well cell culture plate one day before the drug treatment. Cells were treated with tunicamycin (5 μg/ml) for at different time points and washed with 1X phosphate buffered saline (PBS) (Thermo Scientific). Cells were incubated in a lysis buffer (20 mM Tris-HCl (pH 8.0), 80 mM KCl, 1 mM EDTA, 0.5% NP-40, 1 mM DTT, 1X protease inhibitor cocktail and 1X phosphatase inhibitor cocktail) for 10 min and lysed by pipetting followed by vortexing for 20 min at 4°C. The cell extract was centrifuged at 12,000 g for 20 min at 4°C. The clear supernatant containing was collected and subjected to Western blot analysis using appropriate antibody. Human cells used in this study are shown in Table 5.

#### Analysis of biomolecule structures:

The coordinates of the predicted Slt2 structure were retrieved from the AlphaFold website (https://alphafold.ebi.ac.uk) and analyzed using the free PyMol software (https://www.pymol.org/pymol)

#### Densitometry analysis and Statistical Analysis:

The densitometry analysis was performed using the NIH ImageJ gel analysis software[66]. All bands at the correct molecular weight ± approximately 5 kDa were analyzed thrice as the signal for that target protein. The average of three measurements with SD (standard deviation) are shown in each plot.

All quantitative experiments reported in this study (mRNA quantification by qPCR and *LacZ* reporter assays) were conducted using a minimum of three independent biological replicates (n = 3), with some experiments including four or five replicates. For each biological replicate, yeast strains were independently cultured and treated under identical experimental conditions prior to sample collection.

All quantitative data were plotted and statistically analyzed using GraphPad Prism (version 10.1.1). Statistical parameters—including mean, standard deviation (SD), standard error of the mean (SEM), and *P* values—were calculated in GraphPad Prism. Statistical significance was assessed using paired Student’s *t* test, one-way ANOVA, or two-way ANOVA, as appropriate.

## Supplementary Material

1

## Figures and Tables

**Table 1.** List of yeast strains used in this study.

Strain Name	Genotype	Reference
WT (BY4771)	*MAT****a*** *his3-Δ1 leu2-Δ0 met5-Δ0 ura3-Δ0*	Open Biosystems
*ire1Δ*	BY4771-*MAT****a*** *ire1::KanMX*	
*ire1Δ*	BY4771-*MAT****a*** *ire1::HphMx*	This study
*ste20Δ*	BY4771-*MAT****a*** *ste20::KanMX*	
*ste1Δ*	BY4771-*MAT****a*** *ste1::KanMX*	
*ste7Δ*	BY4771-*MAT****a*** *ste7::KanMX*	
*fus3Δ*	BY4771-*MAT****a*** *fus3::KanMX*	
*kss1Δ*	BY4771-*MAT****a*** *kss1::KanMX*	
*pbs2Δ*	BY4771-*MAT****a*** *pbs2::KanMX*	
*hog1Δ*	BY4771-*MAT****a*** *hog1::KanMX*	
*ssk1Δ*	BY4771-*MAT****a*** *ssk1::KanMX*	
*ssk2Δ*	BY4771-*MAT****a*** *ssk2::KanMX*	
*ssk22Δ*	BY4771-*MAT****a*** *ssk22::KanMX*	
*bck1Δ*	BY4771-*MAT****a*** *bck1::KanMX*	
*mkk1Δ, mkk2Δ (mkk1/2Δ)*	BY4771-*MAT****a*** *mkk1::KanMX mkk2:NatMX*	
*slt2Δ*	BY4771-*MAT****a*** *slt2::KanMX*	
*smk1Δ*	BY4771-*MAT****a*** *smk1::KanMX*	
*ire1Δslt2Δ*	BY4771-*MAT****a*** *slt2::KanMX ire1::HphMX*	This study
*rlm1Δ*	BY4771-*MAT****a*** *rlm1::KanMX*	
*smp1Δ*	BY4771-*MAT****a*** *smp1::KanMX*	
*rlm1Δsmp1Δ*	BY4771-*MAT****a*** *rlm1::KanMX smp1::NatMX*	This study
*sir3Δ*	BY4771-*MAT****a*** *sir3::KanMX*	
*rcn2Δ*	BY4771-*MAT****a*** *rcn2::KanMX*	
*caf20Δ*	BY4771-*MAT****a*** *caf20::KanMX*	
*gga1Δgga2Δ*	*MAT****a*** *gga1*Δ::*NAT*^*r*^ *gga2*Δ::*KanMX4*	PMC4786689
*msg5Δ*	BY4771-*MAT****a*** *msg5::KanMX*	
*swi4Δ*	BY4771-*MAT****a*** *swi4::KanMX*	
*swi6Δ*	BY4771-*MAT****a*** *swi6::KanMX*	
*pfk2Δ*	BY4771-*MAT****a*** *pfk2::KanMX*	
*pil1Δ*	BY4771-*MAT****a*** *pil1::KanMX*	
*sod1Δ*	BY4771-*MAT****a*** *sod1::KanMX*	
*tma17Δ*	BY4771-*MAT****a*** *tma17::KanMX*	
*egd1Δ*	BY4771-*MAT****a*** *egd1::KanMX*	
*crz1Δ*	BY4771-*MAT****a*** *crz1::KanMX*	
*ccr4Δ*	BY4771-*MAT****a*** *ccr4::KanMX*	
*gat2Δ*	BY4771-*MAT****a*** *gat2::KanMX*	
*pog1Δ*	BY4771-*MAT****a*** *pog1::KanMX*	
*tec1Δ*	BY4771-*MAT****a*** *tec1::KanMX*	
dig1*Δ*	BY4771-*MAT****a*** dig1*::KanMX*	
*dig2Δ*	BY4771-*MAT****a*** *dig2::KanMX*	
*skn7Δ*	BY4771-*MAT****a*** *skn7::KanMX*	
*msn2Δ msn4Δ*	*MAT****a*** *msn2Δ::hygMX, msn4Δ::KanMX6*	PMC5634782
*kdx1Δ*	BY4771-*MAT****a*** *kdx1::KanMX*	
*WT (W303)*	*MAT****a*** *leu2-3,112 trp1-1 can1-100 ura3-1 ade2-1 his3-11,15} [phi*^*+*^*]*	Lab collection
*rlm1Δ (W303)*	*MAT****a*** *leu2-3,112 trp1-1 can1-100 ura3-1 ade2-1 his3-11,15} [phi*^*+*^*] rlm1::KanMX*	This study
*nas2Δ*	BY4771-*MAT****a*** *nas2::KanMX*	
*nas6Δ*	BY4771-*MAT****a*** *nas6::KanMX*	
*hsm3Δ*	BY4771-*MAT****a*** *hsm3::KanMX*	
*rpn14Δ*	BY4771-*MAT****a*** *rpn14::KanMX*	
*adc17Δ*	BY4771-*MAT****a*** *adc17::KanMX*	
*rpn4Δ*	BY4771-*MAT****a*** *rpn4::KanMX*	
*nas6Δ*	*MATα nas6::TRP*	PMC2727592
*hsm3Δ*	*MATα hsm3::KAN*	PMC2727592
*nas6Δ hsm3Δ*	*MATα nas6::TRP hsm3::KAN*	PMC2727592
*nas6Δ rpn14Δ*	*MATα nas6::TRP rpn14::HYG*	PMC2727592

**Table 2:** List of plasmids used in this study.

Plasmid Name	Plasmid description	Reference or study
D2	pRS314, low-copy Trp3 vector	Lab collection
D3	pRS315, low-copy Leu2 vector	Lab collection
D4	pRS316, low-copy URA3 vector	Lab collection
D8	pRS416, High-copy URA3 Vector	Lab collection
D63	HAC1-WT in D4	Sathe L et al., 2015
D69	HAC1^c^ (intron less) in D4	Uppala et al., 2021
D395	P_Native_Ire1	This study
D1694	HAC1-G771A in D4	Sathe L et al., 2015
D1875	YES1-IRE1 (P_GAL1_Ire1)	This study
D1984	pYES2 vector	Lab collection
D2305	FLAG-SLT2 in D1984	This study
D2716	FLAG-SLT2^1−440^ in D1984	This study
D2720	FLAG-SLT2^1−400^ in D1984	This study
D2313	FLAG-SLT2^1−355^ in D1984	This study
D2276	Human FLAG-ERK1 in D1984	This study
D2277	Human FLAG-ERK2 in D1984	This study
D2760	Human FLAG-ERK5 in D1984	This study
D2255	Human FLAG-ERK5-KD in D1984	This study
D2197	*Schizosaccharomyces pombe* SPK1 in D1984	This study
D2312	Human FLAG-ERK1-D174A in D1984	This study
D2480	Human FLAG-P38α in D1984	This study
D2409	YEp352-RLM1	Dr. Arroyo, University of Madrid, Spain
D2915	P_GAL1_-Rlm1-Flag	This study
D2918	P_GAL1_-Flag-MEF2C	This study
D2805	P_IRE1_-LacZ	This study
D2806	P_IRE1_-ΔRlm1-BS1-LacZ	This study
D2807	P_IRE1_-ΔRlm1-BS2-LacZ	This study
D1421	His_6_-Ire1cyto	Lee et al., 2008
D2808	His_6_-Rlm1^MADX-box^	This study
D2726	Rlm1	Dr. David Levine, Boston University, USA
D2729	Rlm1-S427A	Dr. David Levine, Boston University, USA

**Table 3:** List of antibodies used in this study.

Antibody	Source	Catalog number	Dilution
Anti-BIP1	Santa Cruz Biotechnology	SC-376768	1: 1000
Anti-ATF4	Cell signaling	11815S	1: 1000
Anti-pERK1/2	Cell signaling	9102	1: 1000
Anti-ERK1/2	Invitrogen	13-6200	1: 2000
Anti-GAPDH	Santa Cruz Biotechnology	SC-32233	1: 1000
Anti-Flag	Sigma-Aldrich	F3165	1: 1000
Anti-Slt2	Santa Cruz Biotechnology	SC-133189	1: 1000
Anti- Pgk1	Invitrogen	459250	1:1000
Anti-Hac1	Dey lab -commercial vendor	-	1: 500
Anti-pIRE1	Dey lab-commercial vendor	-	1:10000

**Table 4:** List of primers used for real-time PCR in this study.

Gene	Primers
Yeast IRE1	Forward: 5’-CTTGACACTGCTCGTTTGTGTGT-3’ Reverse: 5’-CGAGACGACAATGGGATTGA-3’
Yeast ACT1	Forward: 5’-CGAAAGATTCAGAGCCCCAGAAGC-3’ Reverse: 5’-CCTTACGGACATCGACATCACAC-3’
Human ATF6	Forward: 5’-ACCCACTAAAGGCCAGACG-3’ Reverse: 5’-CCACGTGATTAGGGAGCTGT-3’
Human IRE1	Forward: 5’-CCAGACAGACCTGCGTAAAT-3’ Reverse: 5’-CCGGTAGTGGTGCTTCTTATT-3’
Human RPLP2	Forward: 5’-CGTCGCCTCCTACCTGCT-3’ Reverse: 5’-CATTCAGCTCACTGATAACCTTG-3’
Human ERK1	Forward: 5’-GCTTCCTGACGGAGTATGTG-3’ Reverse: 5’-CAGATGTCGATGGACTTGGTATAG-3’
Human ERK2	Forward: 5’-AGAGAACCCTGAGGGAGATAAA-3’ Reverse: 5’-CGATGGTTGGTGCTCGAATA-3’
Human ERK5	Forward: 5’-GCCCTTTGACTTTGCCTTTG-3’ Reverse: 5’-CTTGCATGGAAGTCCTCAATTTC-3’
Human MEF2C	Forward: 5’-CCGTAGCAACTCCTACTTTACC-3’ Reverse: 5’-GATGACAGGTCTGCACTACTC-3’
Human HYOU1	Forward: 5’-GCCTGTATTTCTAGACCTGCC-3’ Reverse: 5’-TTCATCTTGCCAGCCAGTTG-3’

**Table 5:** List of human cells used in this study.

Cell lines	Source	Collection
WA09	Wicell Research Institute, Madison, Wisconsin, USA	Gouignard’ lab, UWM
MCF10A cells	ATCC Cat# CRL-10317; RRID: CVCL_0598	Raghavan’s lab, MCW,
Ovarian surface epithelial cells (OSE)	Medical College of Wisconsin	Raghavan’s lab, MCW
HEK293FT	Invitrogen R70007	Dey and An-Phu’s lab
