# Supplementary material for: Coupling the MAPK Slt2/ERK1 Pathway and IRE1-driven UPR Through Transcription Factor Rlm1/MEF2
Source: Res Sq. 2025 Dec 11:rs.3.rs-7292507. Preprint. [Version 1] doi: 10.21203/rs.3.rs-7292507/v1 (PMC12776503; doi:10.21203/rs.3.rs-7292507/v1)
Supplement: 1 [file NIHPPRS7292507V1-supplement-1.pdf]

Supplemental Figures

Figure S1: Yeast cell lacking the MAP kinase Slt2 is sensitive to tunicamycin

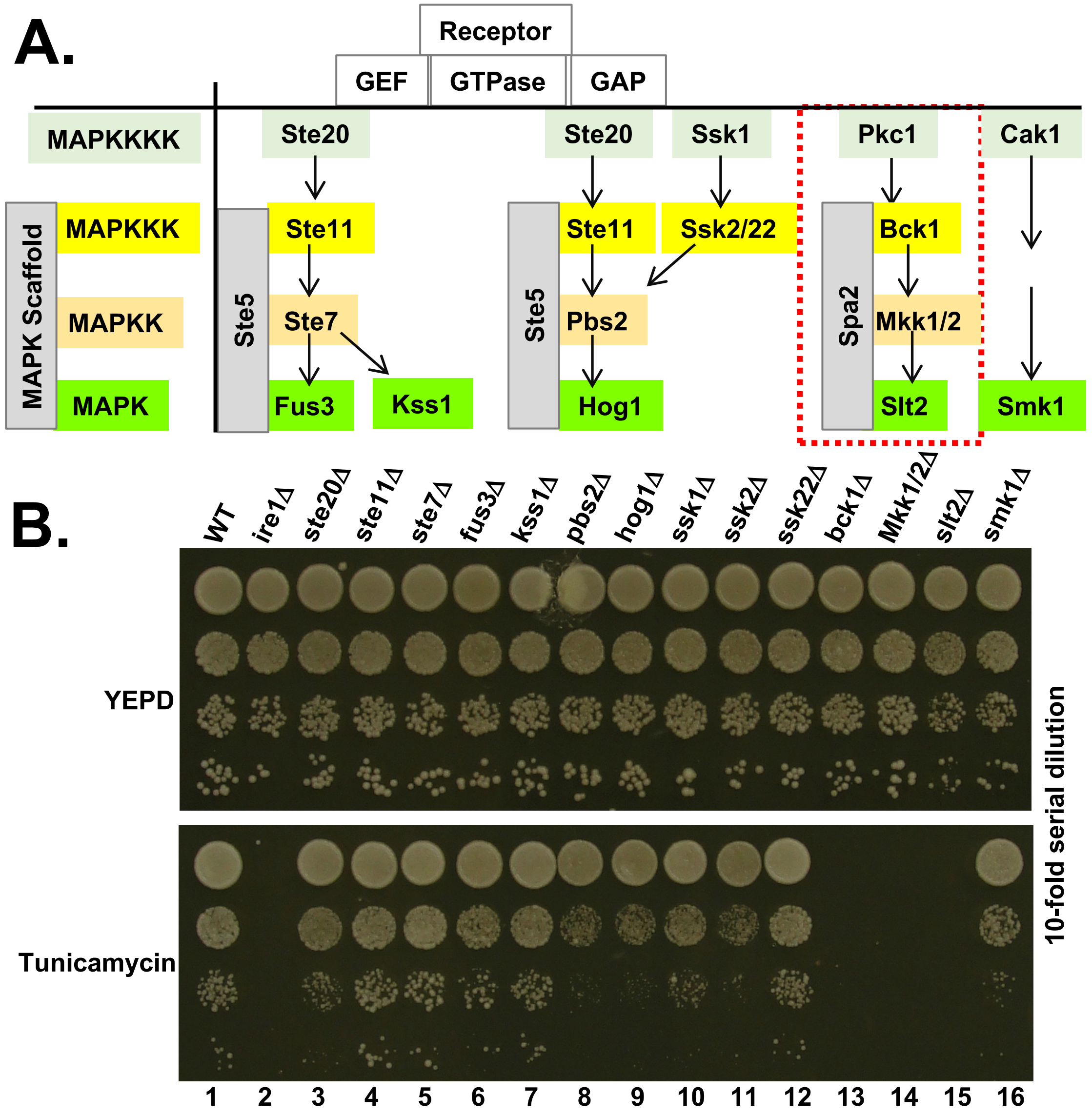

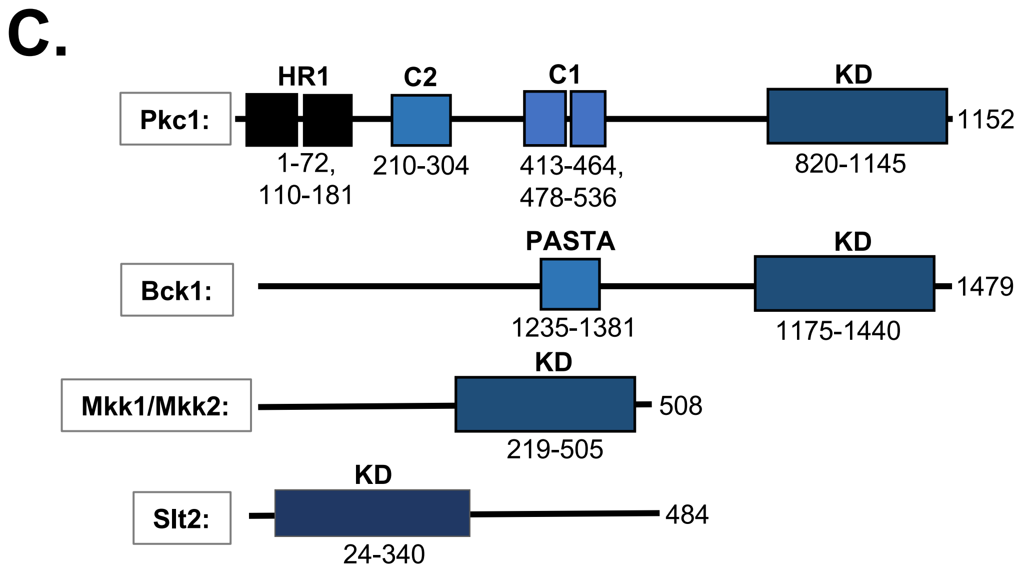

(A) A simplified cartoon representation of the major MAP kinase signaling pathways in yeast *Saccharomyces cerevisiae,* which are regulated by receptors, guanine exchange factor (GEF), GTPase and GTPase activating proteins (GAPs).

(B) The indicated yeast deletion strains were serially diluted, spotted and grown on the YEPD and the same medium containing tunicamycin at 30ºC for 48 hours.

(C) Schematic representation of protein kinases Pkc1, Bck1, Mkk1/Mkk2 and Slt2 with their respective HRI (heme-regulated inhibitor 1) , C1 (conserved homology 1), C2 (conserved homology 2), KD (kinase domain) and PASTA domains. The amino acids making the respective domains are indicated below.

Figure S2: The *ire1Δ slt2Δ* strain is severely sensitive to tunicamycin

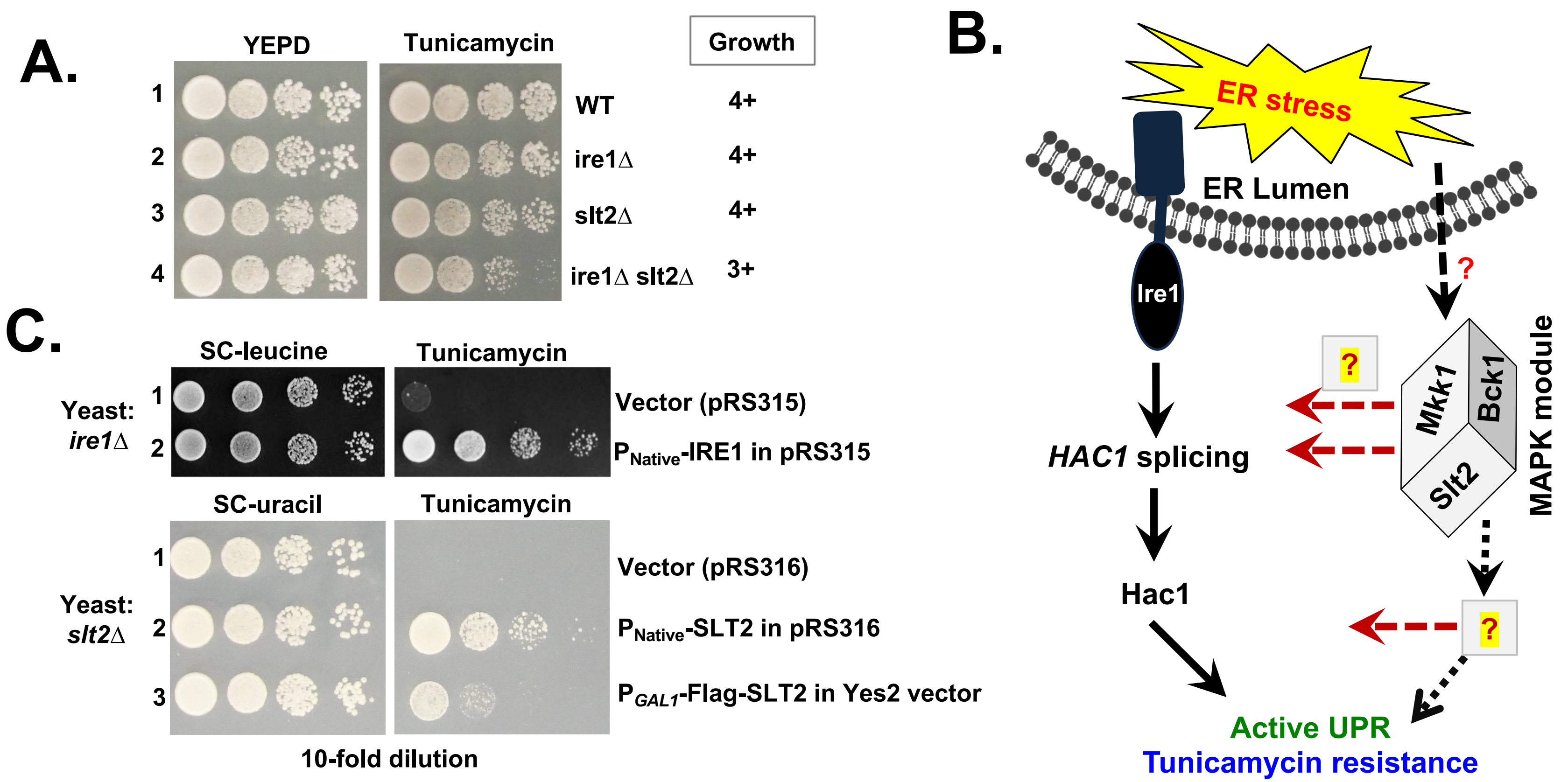

(A) The indicated yeast deletion strains were serially diluted, spotted and grown on the YEPD and the same medium containing tunicamycin (0.1 μg/ml) at 30°C for 48 hours. The grow was measured arbitrarily and indicated.

(B) The schematic representation of Ire1 and the proposed Slt2 pathways in the ER stress response. The unknown links are indicated by red arrows and the sign "?".

(C) The *ire1Δ* or *slt2Δ* strains expressing an IRE1 or SLT2 or Flag-SLT2 protein from its own promoter (P_{Native}-IRE1 in pRS315 or P_{Native}-SLT2 in pRS316) or the *GAL1* promoter (P_{GAL1}-Flag-SLT2 in Yes2 vector) were grown on SC-uracil medium or the same medium containing tunicamycin.

Figure S3: Reduced expression of Hac1 protein in the *slt2Δ* strain

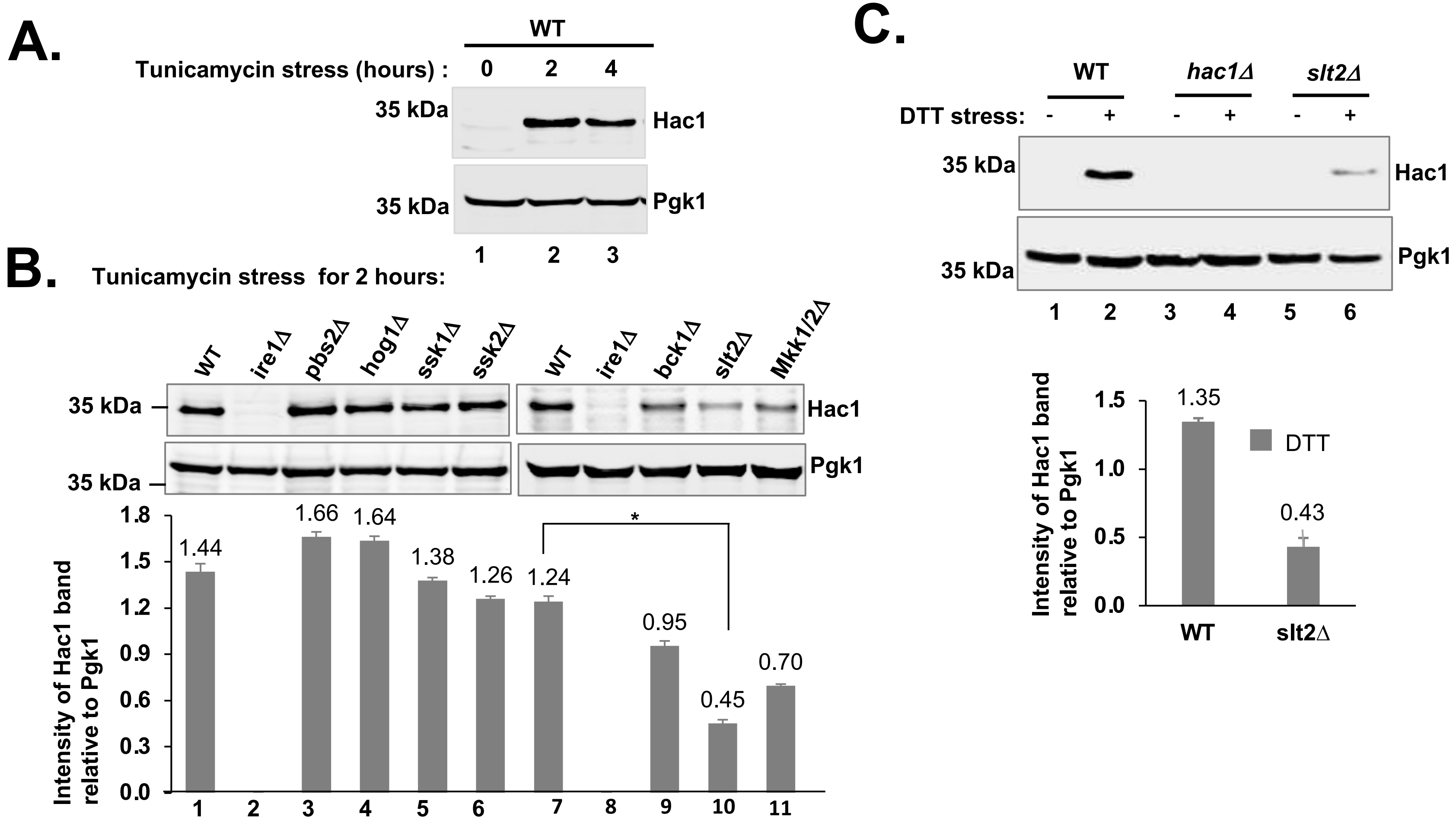

(A) and (B). The indicated yeast deletion strains were grown in the YEPD medium in the absence and presence of tunicamycin. WCEs were prepared and subjected to Western blot analysis using antibodies specific to Hac1 and Pgk1 proteins. The intensities of Hac1 and Pgk1 protein bands were quantified using the ImageJ software. (Bottom panel) The bar diagram depicts the relative intensities of Hac1 protein bands.

(C) The indicated yeast deletion strains were grown in the YEPD medium in the absence and presence of DTT for 2 hours. WCEs were prepared and subjected to Western blot analysis using antibodies specific to Hac1 and Pgk1 proteins. The intensities of Hac1 and Pgk1 protein bands were quantified using the ImageJ software. (Bottom panel) The bar diagram depicts the relative intensities of Hac1 protein bands.

Figure S4: Reduced splicing of HAC1 mRNA in cells lacking MAPK Slt2

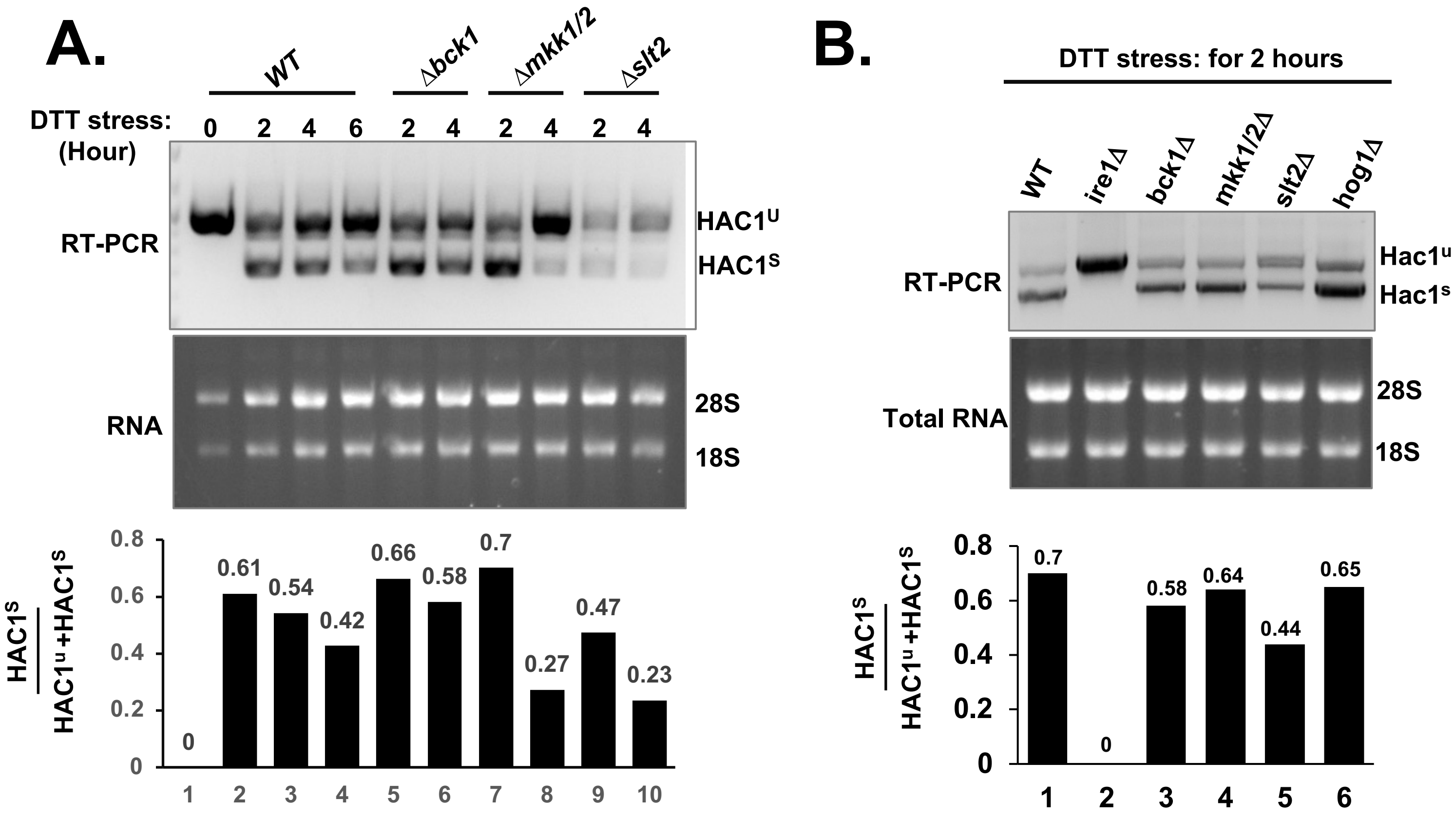

(A) and (B) . The indicated yeast deletion strains were grown in the YEPD medium until they reached the OD_{600} value ~0.5-0.6. Cells were then exposed to DTT (mM) and harvested after 2 hours. Total RNA was extracted and subjected to RT-PCR analysis to distinguish between the spliced ($HAC1^s$) and un-spliced ($HAC1^u$) *HAC1* mRNAs. (Lower panel) The intensities of $HAC1^s$ mRNAs were quantified using the ImageJ software. The bar diagram depicts the relative intensities of $HAC1^s$ mRNAs.

Figure S5: Analysis of the Activation Loop of MAP Kinases

A

```
                                   Catalytic loop           Activation loop    T190 Y192
Slt2            .HRDLKPGNLLVNADCQLKICDFG LARGYSENPVENSQFLTEYVATRWYR APE.
MAPK1(ERK2)     .HRDLKPSNLLLNTTCDLKICDFG LAR-VADPDHDHTGFLTEYVATRWYR APE.
MAPK3(ERK1)     .HRDLKPSNLLINTTCDLKICDFG LAR-IADPEHDHTGFLTEYVATRWYR APE.
MAPK8(JNK1)     .HRDLKPSNIVVKSDCTLKILDFG LAR-----TAGTSFMMTPYVVTRYYR APE.
MAPK9(JNK2)     .HRDLKPSNIVVKSDCTLKILDFG LAR-----TACTNFMMTPYVVTRYYR APE.
MAPK10(JNK3)    .HRDLKPSNIVVKSDCTLKILDFG LAR-----TAGTSFMMTPYVVTRYYR APE.
MAPK11(p38α)    .HRDLKPSNVAVNEDCELRILDFG LAR-----QADEE--MTGYVATRWYR APE.
MAPK12(p38β)    .HRDLKPGNLAVNEDCELKILDFG LAR-----QADSE--MTGYVVTRWYR APE.
MAPK13(p38γ)    .HRDLKPGNLAVNEDCELKILDFG LAR-----HADAE--MTGYVVTRWYR APE.
MAPK14(p38δ)    .HRDLKPSNLAVNEDCELKILDFG LAR-----HTDDE--MTGYVATRWYR APE.
```

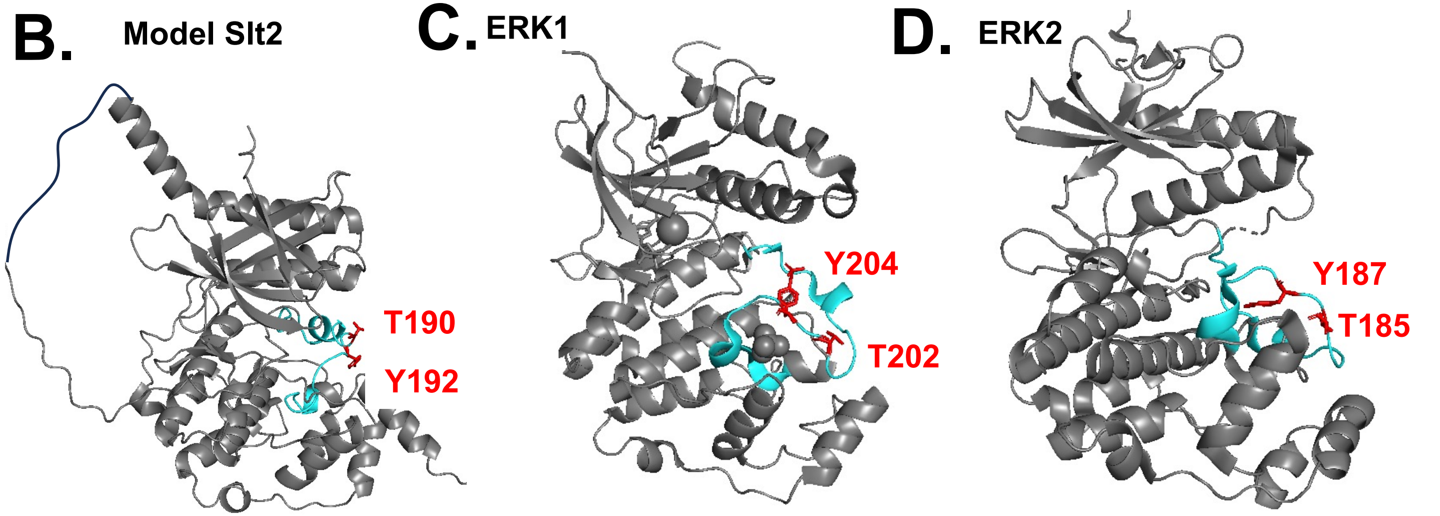

A. Sequence alignment of Slt2 with other indicated MAP kinases. The catalytic and activations loop regions are shown. The phosphorylated threonine and tyrosine residues of Slt2 are marked in red.
B. The cartoon of Slt2 AlphaFold structure is shown, with the activation loop highlighted in cyan. The phosphorylated threonine and tyrosine residues are marked in red.
C. & D. The cartoon of ERK1 and ERK2 structures (PDB = 2zoq and , respectively) is shown, with the activation loop highlighted in cyan. The phosphorylated threonine and tyrosine residues are marked in red.

Figure S6: Analysis of the Ire1 Activation loop in its oligomeric structure

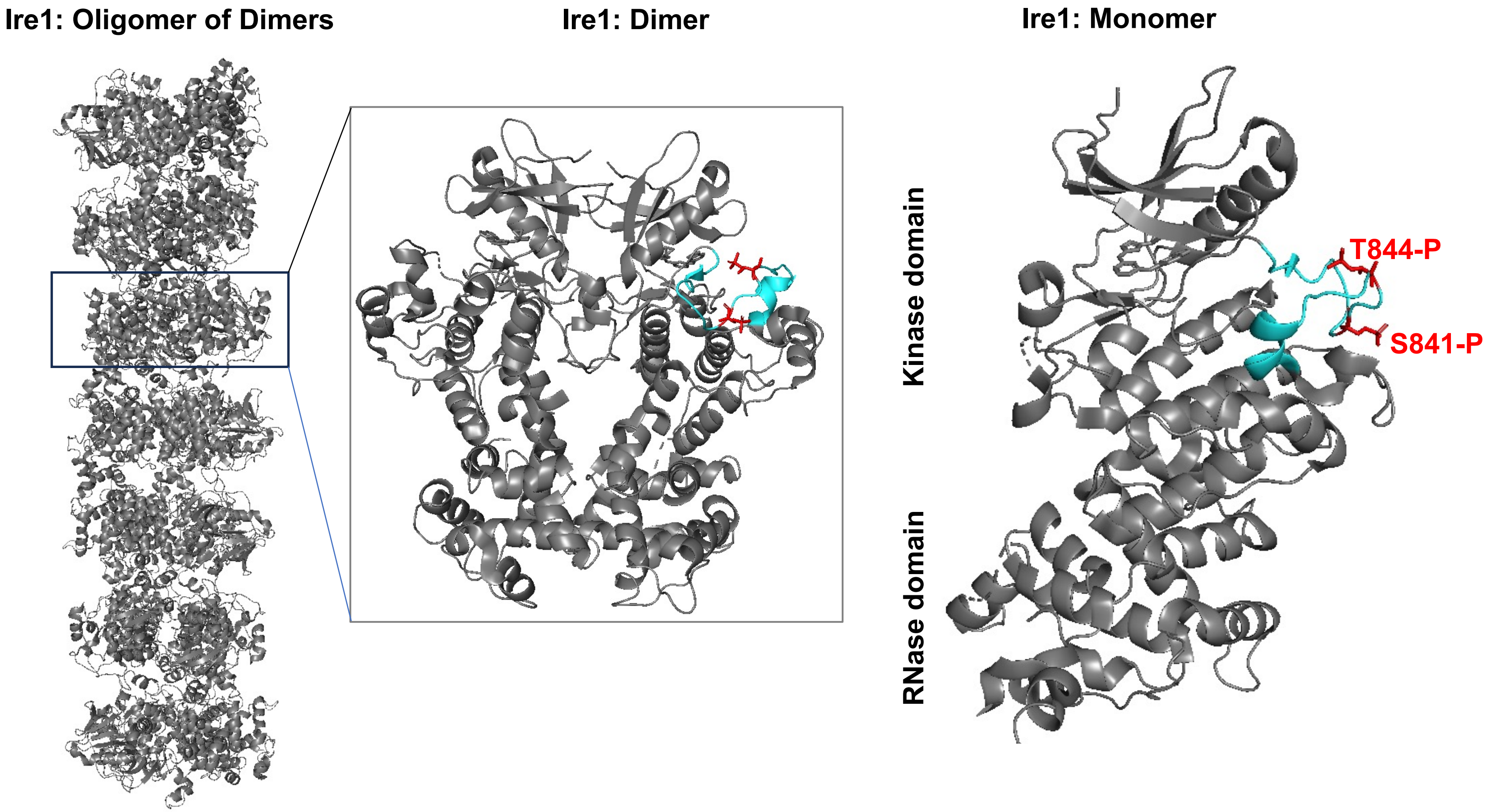

The PyMol software was used to analyze the oligomeric structure of Ire1 cytoplasmic domain (PDB ID = 3fbv). The activation loop of the monomer is shown in cyan, with the phosphorylated residues marked in red.

Figure S7: Kdx1 is a Pseudokinase of MAPK Slt2

A.

```
                                    P-loop
Slt2    MADKIERHTFKVFNQDFSVDKRFQLIKEIGHGAYGIVCSARFAEAAEDTTVAIKKVTNVF 60
Kdx1    MATDTERCIFRAFGQDFILNKHFHLTGKIGRGSHSLICSSTYTESNEETHVAIRKIPNAF 60
        ** . **  *:.*.*** ::*:*:*  :**:*::.::**: ::*: *:* ***:*: *.*

Slt2    SKTLLCKRSLRELKLLRHFRGHKNITCLYDMDIVFYPDGSINGLYLYEELMECDMHQIIK 120
Kdx1    GNKLSCKRTLRELKLLRHLRGHPNIVWLFDTDIVFYPNGALNGVYLYEELMECDLSQIIR 120
        .:.* ***:*********:*** **. *:* ******:*::**:**********: ***:
                                  Catalytic loop
Slt2    SGQPLTDAHYQSFTYQILCGLKYIHSADVLHRDLKPGNLLVNADCQLKICDFGLARGYSE 180
Kdx1    SEQRLEDAHFQSFIYQILCALKYIHSANVLHCDLKPKNLLVNSDCQLKICNFGLSCSYSE 180
        * * * ***:*** *****.*******:*** **** *****:*******:***: .***
          Activation loop
Slt2    NPVENSQFLTEYVATRWYRAPEIMLSYQGYTKAIDVWSAGCILAEFLGGKPIFKGKDYVN 240
Kdx1    NHKVNDGFIKGYITSIWYKAPEILLNYQECTKAVDIWSTGCILAELLGRKPMFEGKDYVD 240
        *   *. *:. *::: **:****:*.**  ***:*:**:******:** **:*:*****:
Slt2    QLNQILQVLGTPPDETLRRIGSKNVQDYIHQLGFIPKVPFVNLYPNANSQALDLLEQMLA 300
Kdx1    HLNHILQILGTPPEETLQEIASQKVYNYIFQFGNIPGRSFESILPGANPEALELLKKMLE 300
        :**:***:*****:***:.*.*::* :**.*:* **   * .: *.** :**:**::**
Slt2    FDPQKRITVDEALEHPYLSIWHDPADEPVCSEKFEFSFESVNDMEDLKQMVIQEVQDFRL 360
Kdx1    FDPKKRITVEDALEHPYLSMWHDIDEEFSCQKTFRFEFEHIESMAELGNEVIKEVFDFRK 360
        ***:*****::********:***  :*  *.:.*.*.** ::.* :* : **:** ***
Slt2    FVRQPLLEEQRQL-QLQQQQQQQQQQQQQQQQPSDVDNGNAAASEENYPKQMATSNSVAP 419
Kdx1    VVRKHPISGDSPSSSLSLEDAIPQ--EVVQVHPSRKV-LPSYSPEFSYVSQLPSLTTTQP 417
        .**:  :. :    .*. ::   *  :  * :**      : : * .* .*: : .:. *
Slt2    QQESFGIHSQNLPRHDADFPPRPQESMMEMRPATGNTADIPPQNDNGTLLDLEKELEFGL 479
Kdx1    YQNLMGISSNSFQGVN*------------------------------------------- 433
         *: :** *:.:   :
Slt2    DRKYF* 484
Kdx1    ------ 433
```

E = 2e-168, Identity = 53%, Positive = 68%

B.

Motif	Function	Kinase	
		Slt2	Kdx1
$G_xG_{xx}G$	Anchors the β-PO_4 of ATP	GHGAYG	GRGSHS
VAIK	Anchors both α- and β-PO_4 of ATP	VAIK	VAIR
HRD	Catalytic base	HRD	HCD
DFG	Chelates Mg^{2+}	DFG	NFG
APE	Stabilizes the activation loop	APE	APE

C.

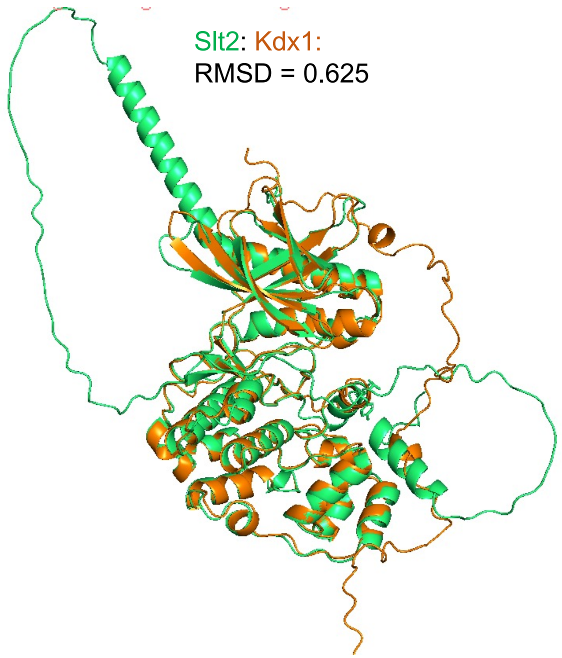

D.

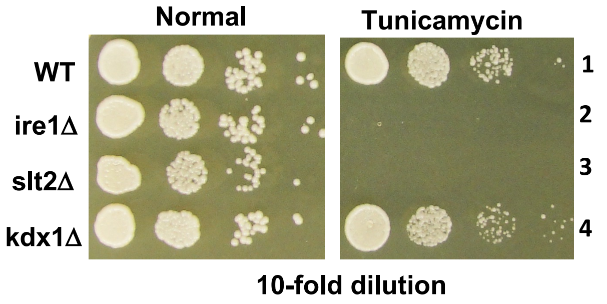

(A) Protein sequence alignment of Slt2 and its psudokinase Kdx1. The conserved motifs and residues in the P-loop, catalytic loop and the activation loop are highlighted in red.
(B) Comparison of conserved protein motifs in Slt2 and Kdx1.
(C) Superimposition of Alpha-fold predicted structures of Slt2 (green) and Kdx1 (orange).
(D) The indicated yeast deletion strains were serially diluted, spotted and grown on the YEPD and the same medium containing tunicamycin at 30°C for 48 hours.

Figure S8: Known substrates of MAPK Slt2

A.

	**Slt2 substrates**
**Transcription & related**	Transcription factor **Rlm1**
	RNA polymerase II subunit (**RbpII-CTD**)
	Transcription factor BTF3 homolog **Egd1**
	Transcriptional regulator **SBF**
	Silencing protein **Sir3**
	Calcineurin regulator **Rcn2**
**Translation & related**	Translation repressor protein **Caf20**
	The Golgi-associated adaptor **Gga1**
**Protein kinase & related**	MAPK **Mkk1**
	PKA regulatory subunit **Bcy1**
	Cyclin C of the Cdk8/**Cyc8**
**Phosphatase & related**	Phosphofructokinase **Pfk2**
	Phosphatase **Msg5**
**Chaperones**	ATPase & chaperone **Tma17**
	Eisosome core component **Pil1**
	Superoxide dismutase **Sod1**

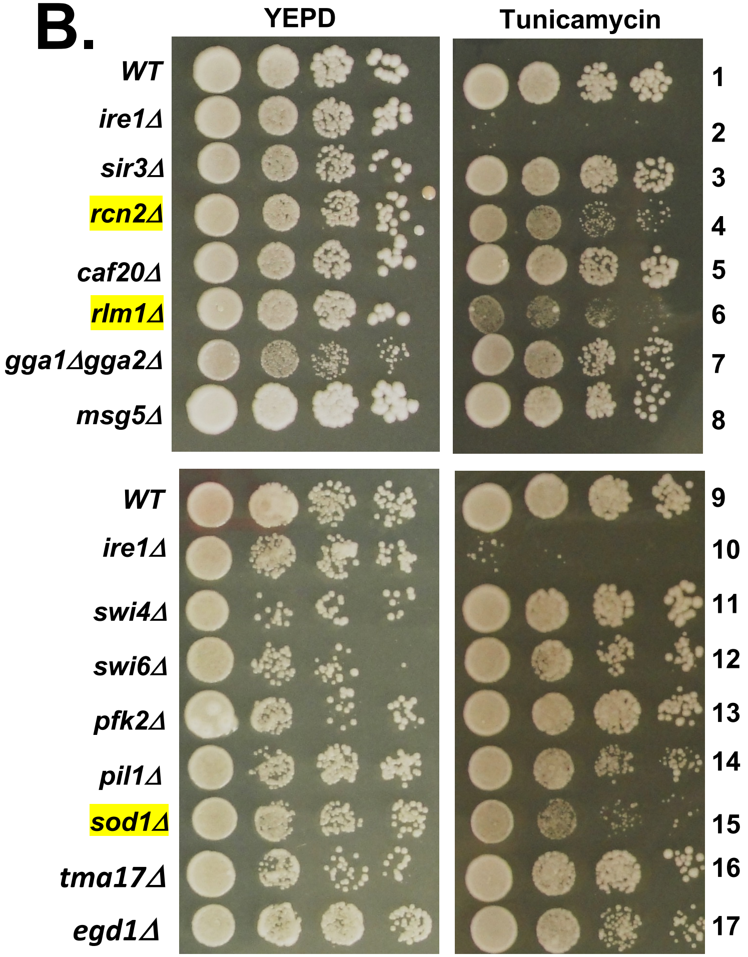

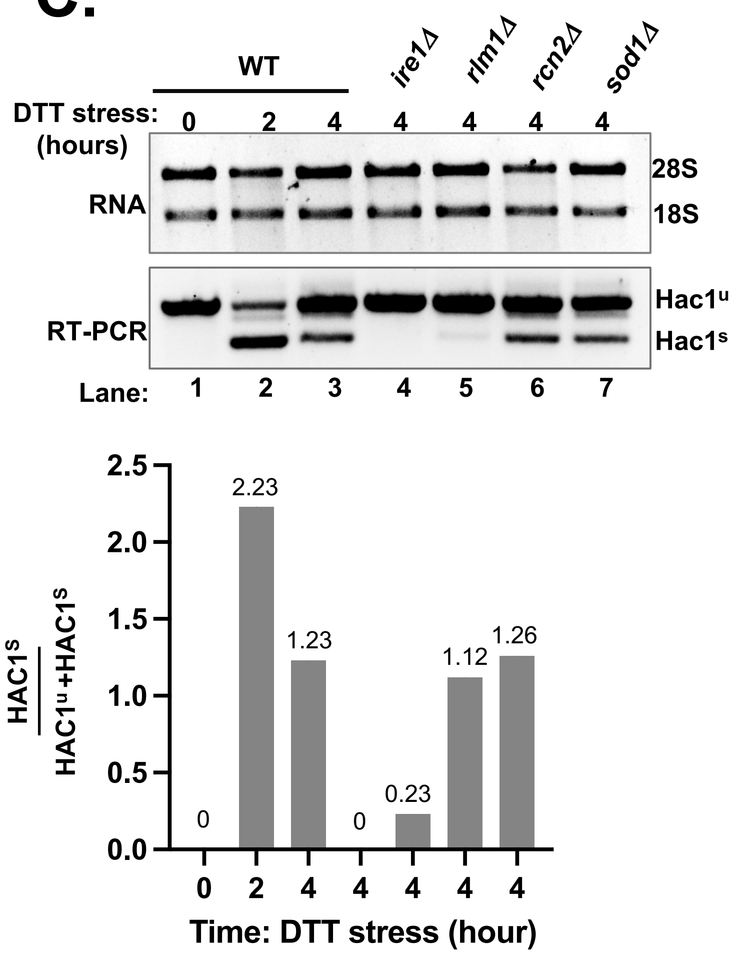

(A) List known Slt2 substrates.
(B) The indicated yeast deletion strains were serially diluted, spotted and grown on the YEPD and the same medium containing tunicamycin at 30ºC for 48 hours.
(C) The indicated yeast deletion strains were grown in the YEPD medium until they reached the OD_{600} value ~0.5-0.6. Cells were then exposed to DTT (5 mM) and harvested after 4 hours. Total RNA was extracted and subjected to RT-PCR analysis to distinguish between the spliced ($HAC1^s$) and un-spliced ($HAC1^u$) *HAC1* mRNAs

Figure S9: Physical Interactomes of Kdx1

A.

	Gene	Paralog	Known Function
Transcription factor	CCR4		Transcriptional complex factor
	CRZ1		Transcription factor
	DIG1	DIG2	Transcriptional activator
	GAT2		Transcriptional activator
	MSN2	MSN4	Transcriptional activator
	POG1		Transcriptional activator
	STE12		Transcription factor
	TEC1		Transcription factor
	RLM1	SMP1	Transcription factor
	SKN7	HSM2	Transcription factor
Protein kinase	BCK1		Serine/threonine protein kinase
	CDC28		Cyclin-dependent kinase catalytic subunit
	KIN2	KIN1	Serine/threonine protein kinase
	KSP1		Serine/threonine protein kinase
	KSS1		Mitogen-activated protein kinase
	MKK2	MKK1	Serine/threonine protein kinase
	PHO85		Cyclin-dependent kinase
	TOR1	TOR2	Lipid kinase
Phosphatase	MSG5	SDP1	Dual specificity phosphatase
ATpase	ISW1		Chromatin remodeler
Others	SAH1		S-adenosyl-L-homocysteine hydrolase
	MPT5		mRNA-binding protein of the PUF family
	YPD1		Osmotic stress-responsive protein
	RGC2	RGC1	Activator of SKn7 transcription factor (ASK10)
	GFA1		Amidotransferase

B.

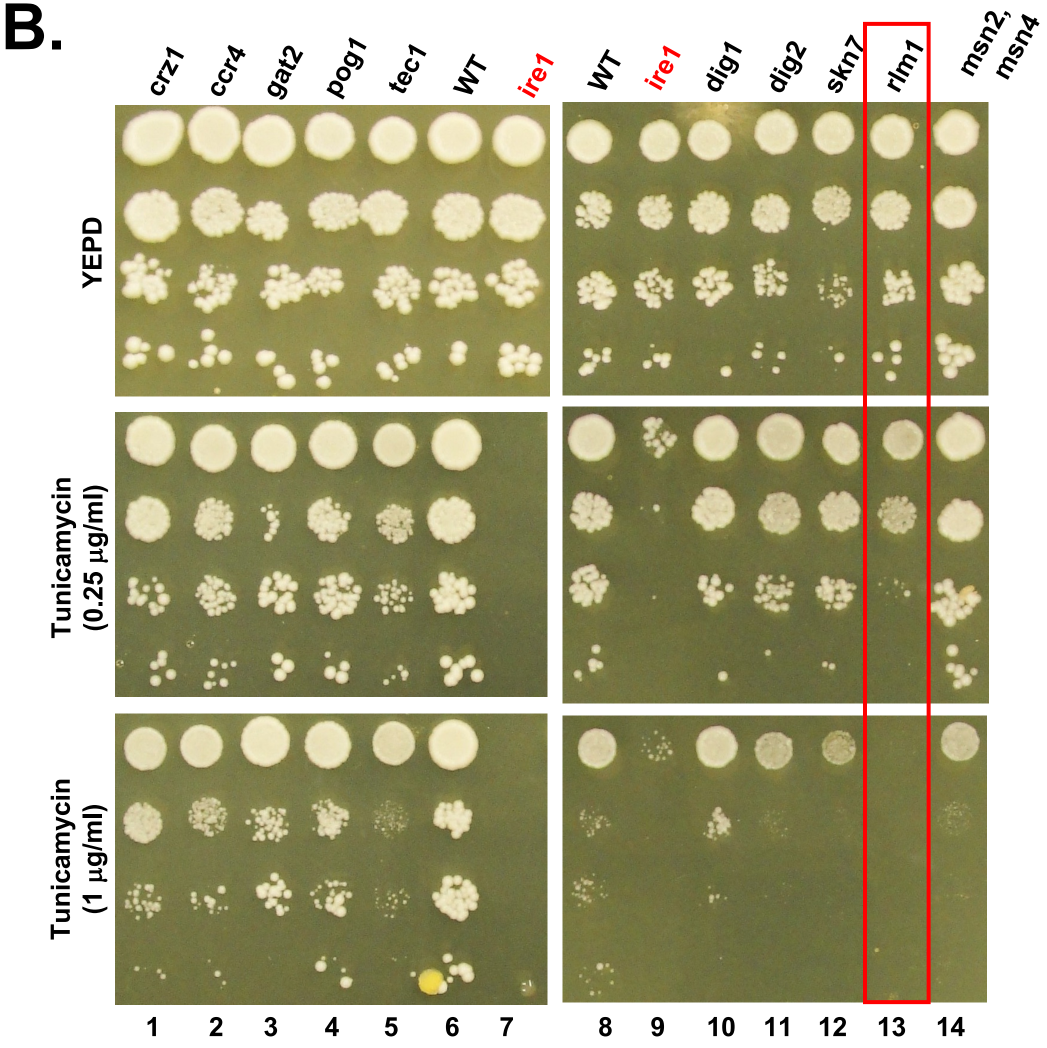

(A) List of known interacting partners of Kdx1.

(B) The indicated yeast deletion strains were serially diluted, spotted and grown on the YEPD and the same medium containing tunicamycin at 30°C for 48 hours.

Figure S10: Reduced splicing of HAC1 mRNA in cells lacking Rlm1

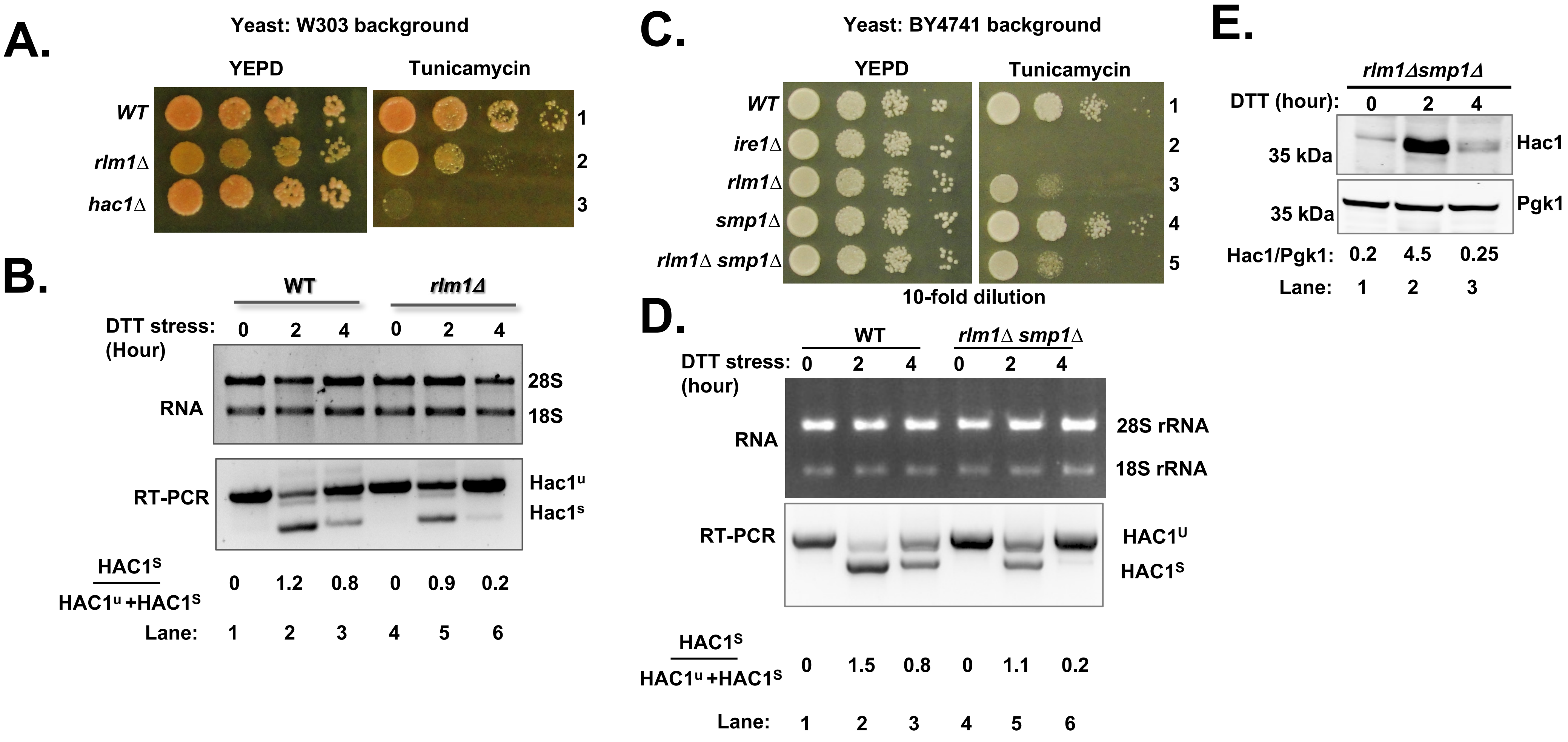

(A) The indicated yeast deletion strains were serially diluted, spotted and grown on the YEPD and the same medium containing tunicamycin at 30°C for 48 hours.

(B) The indicated yeast deletion strains were grown in the YEPD medium until they reached the OD_{600} value ~0.5-0.6. Cells were then exposed to DTT (5 mM) and harvested after indicated time. Total RNA was extracted and subjected to RT-PCR analysis to distinguish between the spliced ($HAC1^s$) and un-spliced ($HAC1^u$) *HAC1* mRNAs

Figure S11: Comparative protein sequence analysis of Rlm1 and MEF2 transcription factors

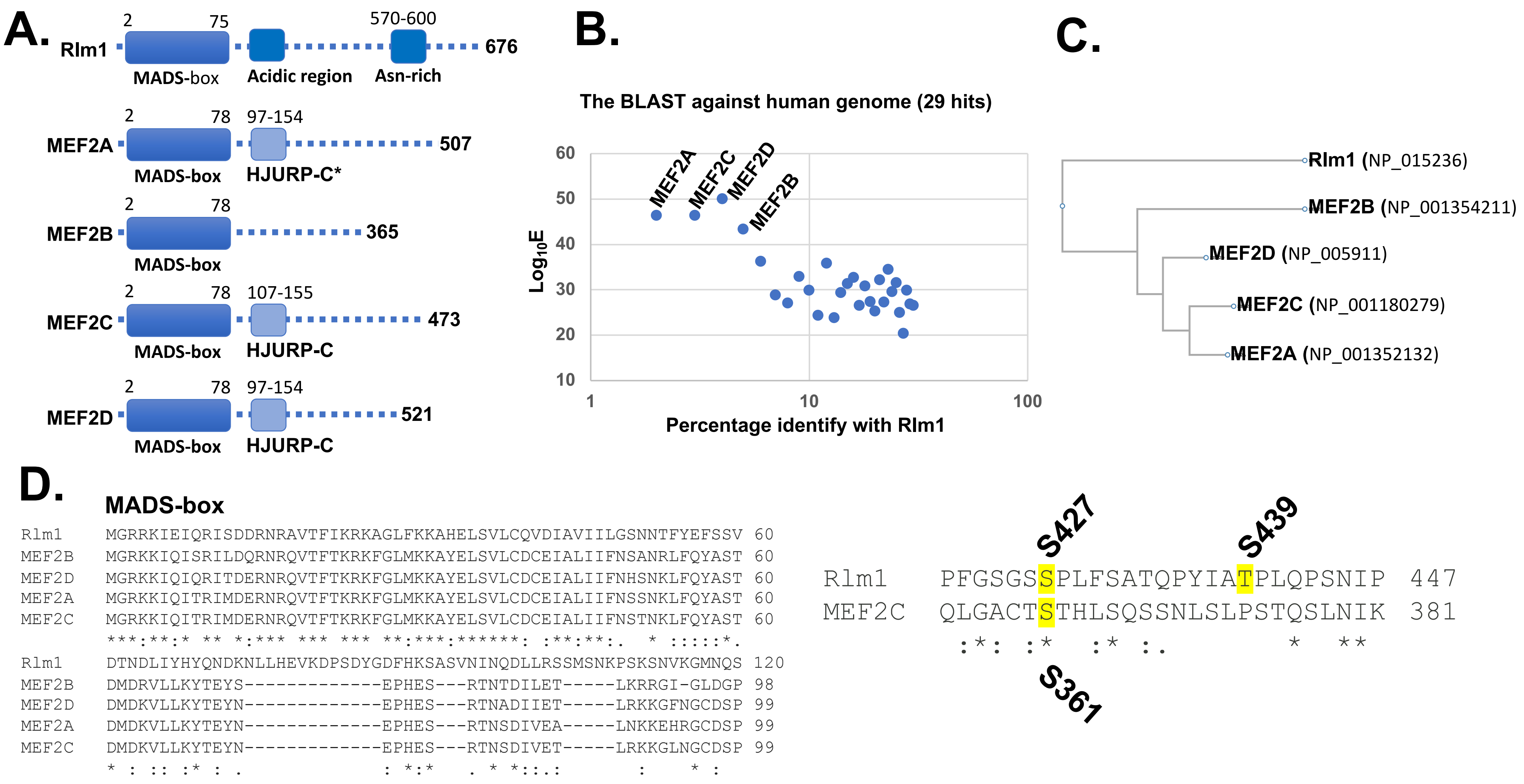

A. Schematic diagram of Rlm1, MEF2A, MEF2B, MEF2C and MEF2D proteins. The protein domains are indicated by rectangular boxes and amino acids are indicated by numbers. HJURP-C*: Holliday junction regulator protein family C-terminal repeat
B. A BLAST search was conducted using the Rlm1 sequence against the human genome. The percentage identify and Log-E-values of the BLST output are plotted and shown.
C. Dendrogram shows the relationship between Rlm1 and MEF2 family member transcription factors. The accession number of proteins are shown in brackets.
D. Rlm1 (NP_015236), MEF2A (NP_001352132), MEF2B (NP_001354211), MEF2C (NP_001180279) and MEF2D ((NP_005911) were aligned using Clustal omega. ((Left panel)The alignment of the MADS box domains is only shown. (Right panel) S427 in Slt2 corresponds to S361 in MEF2C.

Figure S12: **Expression of Rlm1 or MEF2C from a GAL1 Promoter**

A.

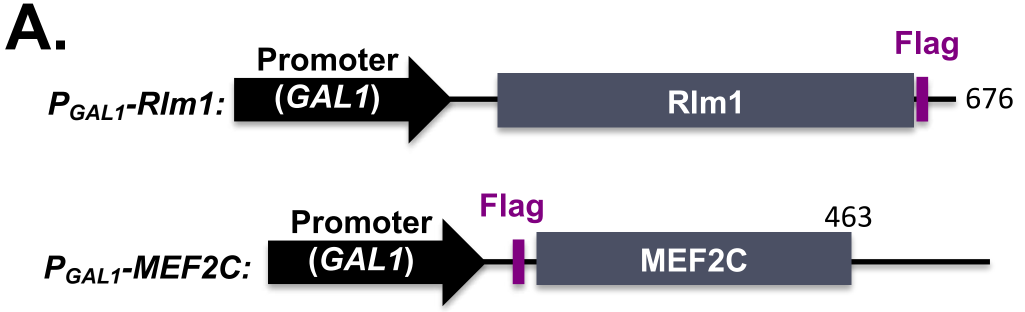

B.

MEF2C Protein Sequence (Acc # NP_001124477)

```
MGRKKIQITR IMDERNRQVT FTKRKFGLMK KAYELSVLCD CEIALIIFNS TNKLFQYAST
DMDKVLLKYT EYNEPHESRT NSDIVEALNK KENKGCESPD PDSSYALTPR TEEKYKKINE
EFDNMIKSHK IPAVPPPNFE MPVSIPVSSH NSLVYSNPVS SLGNPNLLPL AHPSLQRNSM
SPGVTHRPPS AGNTGGLMGG DLTSGAGTSA GNGYGNPRNS PGLLVSPGNL NKNMQAKSPP
PMNLGMNNRK PDLRVLIPPG SKNTMPSVNQ RINNSQSAQS LATPVVSVAT PTLPGQGMGG
YPSAISTTYG TEYSLSSADL SSLSGFNTAS ALHLGSVTGW QQQHLHNMPP SALSQLGACT
STHLSQSSNL SLPSTQSLNI KSEPVSPPRD RTTTPSRYPQ HTRHEAGRSP VDSLSSCSSS
YDGSDREDHR NEFHSPIGLT RPSPDERESP SVKRMRLSEG WAT*
```

A. Schematic representation of GAL1-driven Rlm1 and MEF2C constructs
B. MEF2C protein sequence

Figure S13: Partial rescue of UPR activity in the *rlm1Δ* strain

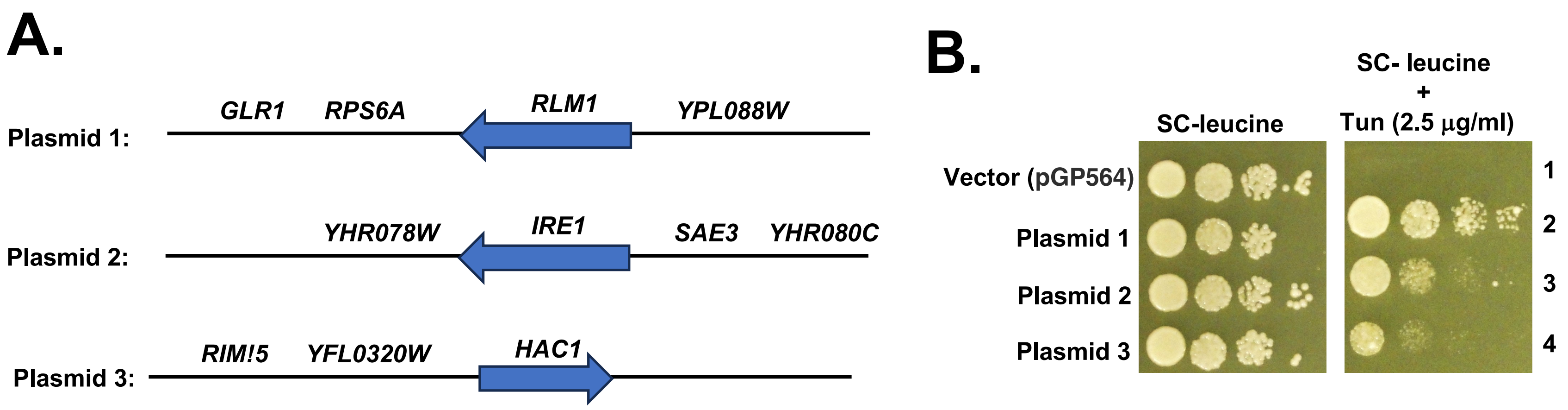

A. The schematic representation of plasmids harboring the indicated open reading frames (ORFs).

B. The *rlm1Δ* strains containing a vector plasmid pGP564 or the indicated plasmids were tested for growth on the SC-leucine medium or the same medium containing tunicamycin.

Figure S14: The potential Rlm1 binding sites in IRE1 and SLT2 promoters

A.

Rlm1 Binding Site (RBS):
Consensus motif

5'-CTA[T/A]$_4$TAG-3'

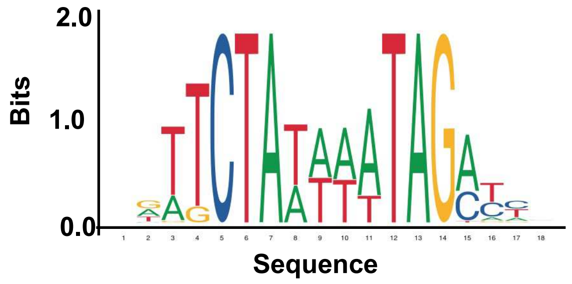

B. **IRE1 promoter sequence**

```
GTCTTATCCT TGCCATAAAC AGAAGACAAC TTGCAAGTAT GAACTATTTG GAAACACAGT TAAATAAAAA GCAAAAACAG ATACAGGAAT ACGAAAGTAT
GAATGGCAAC CTGATAAAGA TGTTTGAGCA ATTGTCTAAA GAAAAGAAAA AGTATGTAGC TATTTTTTCC AGTCGGCAAA AATCGGTATA ACAAACAAAA
AATATTTAGT TTCTGTTATT AACAGAACTT GTCAAAGTGA TGAGACACCA AAAAAAATTT CCTCGACGTA CATTAAAGAG TTAAAGGAGT ACAACGAATT
GAGAGATGCC GGTTTAAGGT TGGCCCAAAT AATTGCTGAT GAAAAGCAAT GCAAAATTAA GGATGTTTTT GAAGAGATCG GTTATTCAAT GAAGGACTAA
TGGGCTTTTA GGGACAGTTC TATTCTTCCA ACGTGCGAAG CGTTCCAATG AAACTCTGCT GCGCGCTGAA AACTATATAA AAAAAAAAAA AGATAAAATT
ACAAAGAAGT AATGAACTTA AATGCTATTA TACAGTTACT AATTAAGCTG CGTAGTGTGA AATATATTGA TAGTTGTAGT TTTGAGGTTT TATTACCCTC
TGTTATATAA TAGGTTTTCG CTATTTTATT GCCGAAAATC AAGACGGAGC GTAAGCCTCT TCGGGCAATA CCTTCGACTA TTCCAACAAT AAAATTTAAA
AAAAGAGATT AATCACATAG TAACAAGAAA TAAACGAAAA ACATATCATA GGAGATCAAT GAGCCAACTT CACATAAACT AACAGTGAAA AGTCTATAAC
AATATTAATT TTACACAATT AAATCTACAC TATAACTGGC ACTGTTAATA ATCGGTAATC TGCTAAGTAA CTTTTTTTTC TCATTCACAA AGCATCGTTT
TCCTCTTCCC CACGTCCATT ATCACTTTTC TCCATATCAC CCTTCATACA CATTAAAAAA ACAGCATATC TGAGGAATTA ATATTTTAGC ACTTTGAAAA ATG
```

C. **SLT2 promoter sequence**

```
TGAAAGAGTA AAAGATTTCT ATAAACGTTT AGAAACTTTG AAATGACTTG CAACGAATAA ATGCATATAC TCTAGTTGAA GTTTTCTTTT CTTGTTCTAT
ACAGGTTCGA ATACTTGTGA GCCTATCTGT ATAATTTAAC AGAATCCCGA AATATTCATC TAGAAGCCAT CTATTTAGCT AAGCCTACGT ATGCGGCGAT
TTTTATATTA TCTTTTTTTT TTTTTATAGA AGACTGCGAA ATGTTGGCAG AATGGAAAGT TTCAGTGTTA AAAATAGAAA CTGAAAAAGG AGATCTAGCC
AGGAATATAT CGAAAAAAAA AGTGAGGGAA ATCAGATCCT ACACAAATAT TTAGATTTAA TTGAAGACCC TGGTCTGCCA GATATATATA TATATTAGAC
GAACTGTGCA TTCAGTCAGC AAATCTAGGC CACAGATTTT CTTATTGAAG CTATCAAAAT AGTAGAAATA ATTGAAGGGC GTGTATAACA ATTCTGGGAG ATG
```

A. Sequence logo of consensus motif of Rlm1 binding site generated from (http://jaspar.genereg.net)
B. The IRE1 promoter sequence, with the predicted Rlm1 binding sites are highlighted.
C. The Slt2 promoter sequence. With the Rlm1 binding site is highlighted.

Figure S15: Structural comparisons of Slt2, ERK1 and ERK2

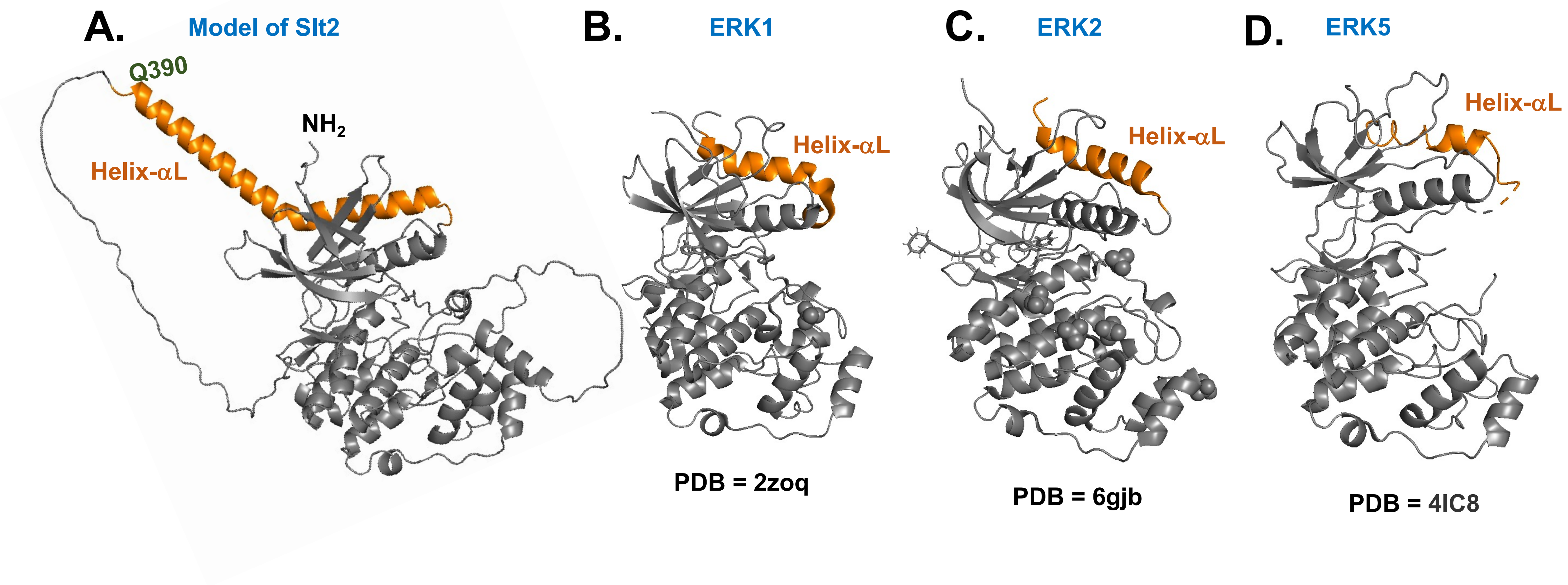

The PyMol software was used to analyze the model structure of Slt2 as determined by AlphaFold as well as crystal structures of ERK1, ERK2 and ERK5. The kinase domain is colored in grey while the helix-αL in orange.

Figure S16: The predicted helix-αL requires for Slt2 function during ER stress response.

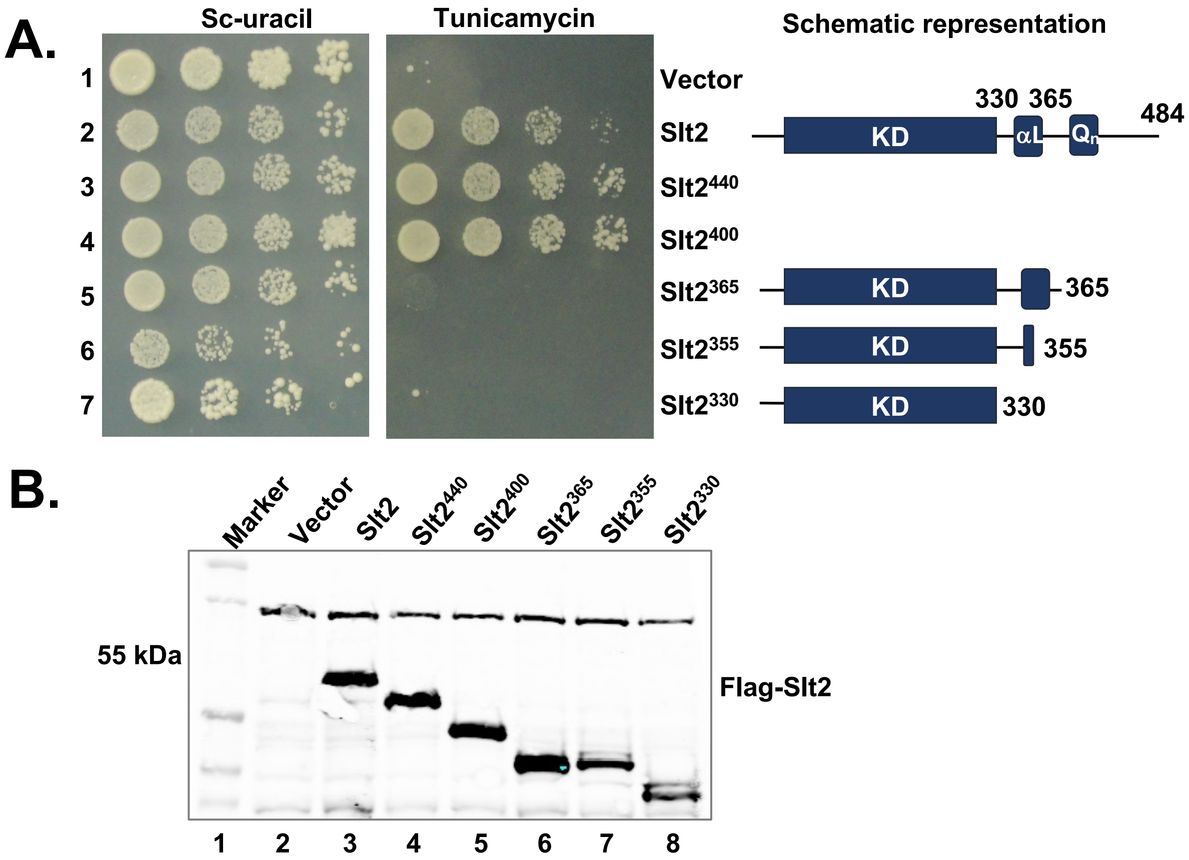

(A) The *slt2Δ* strain containing a Yes2 vector plasmid and the same vector expressing the indicated Flag-tagged Slt2 proteins were tested for growth on the complete synthetic (SC) medium without uracil and the same medium containing tunicamycin.

(B) WCEs were prepared from yeast strain indicated in the panel (D) and subjected to Western blot analysis using antibody specific to the Flag epitope.

Figure S17: The predicted helix-αL requires for Slt2 function during the ER stress response.

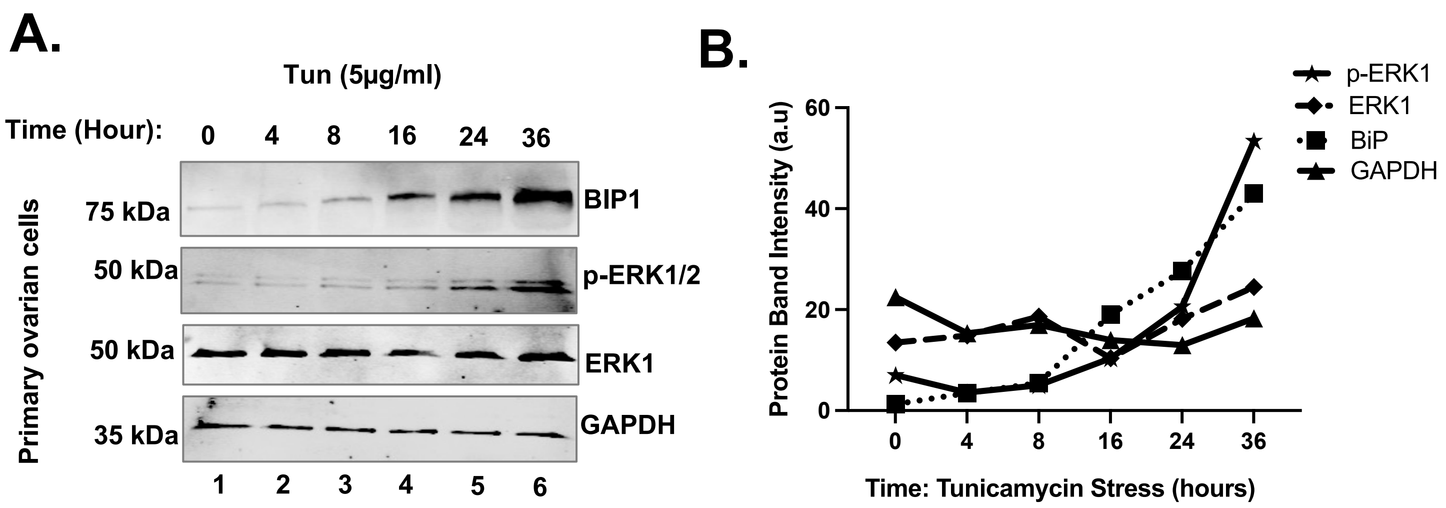

(A) WCEs were prepared from primary ovarian cells after tunicamycin treatment and subjected to Western blot analysis to detect Bip1, pERK1/2, ERK1. and GAPDH .

(B) The protein band intensities are indicated in a line diagram.

Figure S18: Yeas cells lacking components of proteosomes grow on the tunicamycin medium.

A

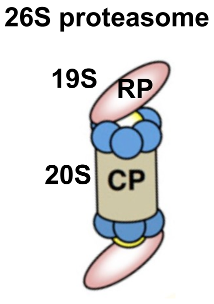

Chaperones for RP

Yeast	Mammal
Nas2	p27
	p28
Hsm3	S5b
Rpn14	Rpn14
Adc17	-

B

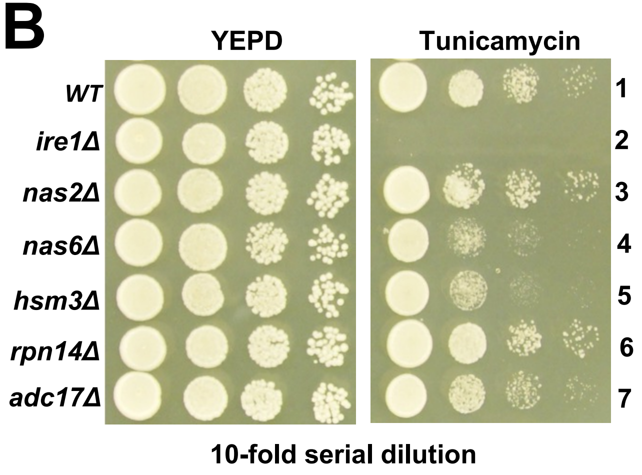

C

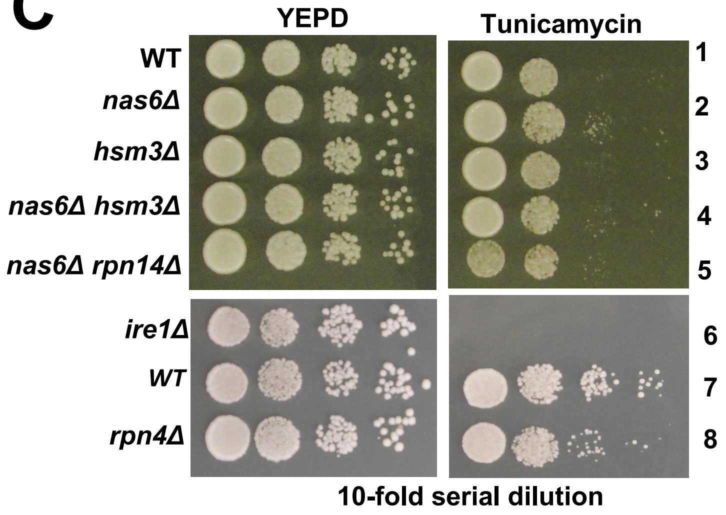

A. (Left panel) The cartoon of 26S proteosome composed of 20S core particle (CP) and 19S regulatory particle (RP). (Right panel) The list of chaperone for the RP are in yeast and mammalian cells.

B. & (C) The indicated yeast deletion strains were serially diluted, spotted and grown on the YEPD and the same medium containing tunicamycin at 30°C for 48 hours.

Figure S19: Complementation of Slt2 null strain by human MAP kinases ERK5

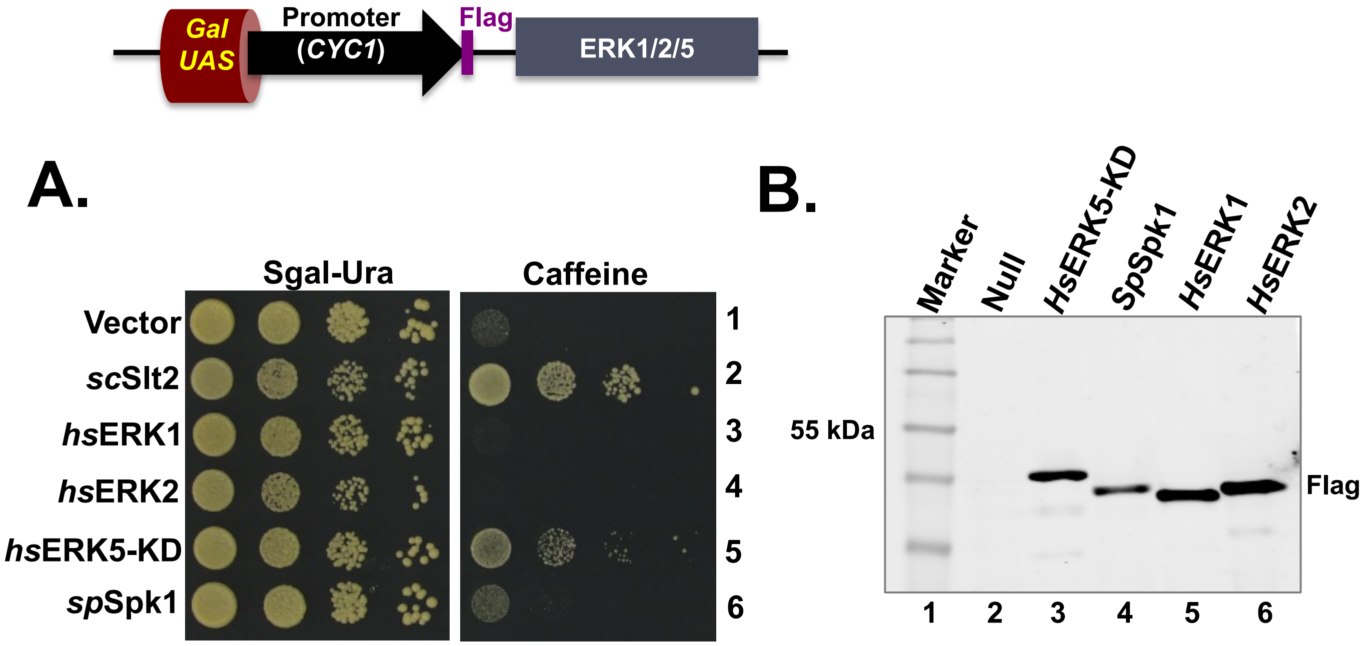

(A) The *slt2Δ* strain containing a vector plasmid or the same vector expressing the indicated Flag-tagged MAP kinases (scSlt2 = Saccharomyces cerevisiae Slt2; *hs*ERK1,2,5 = *Homo sapiens* ERK1, 2, 5; and spSpk1 = *Schizosaccharomyces* pombe Spk1) were tested for growth on the complete synthetic (SC) medium with galactose without uracil and the same medium containing Caffeine.

(B) WCEs were prepared from yeast strain indicated in the panel A and subjected to Western blot analysis using antibody specific to the Flag epitope.

Figure S20: The ERK1 phosphorylation level is high in immortalized HEK293 cells

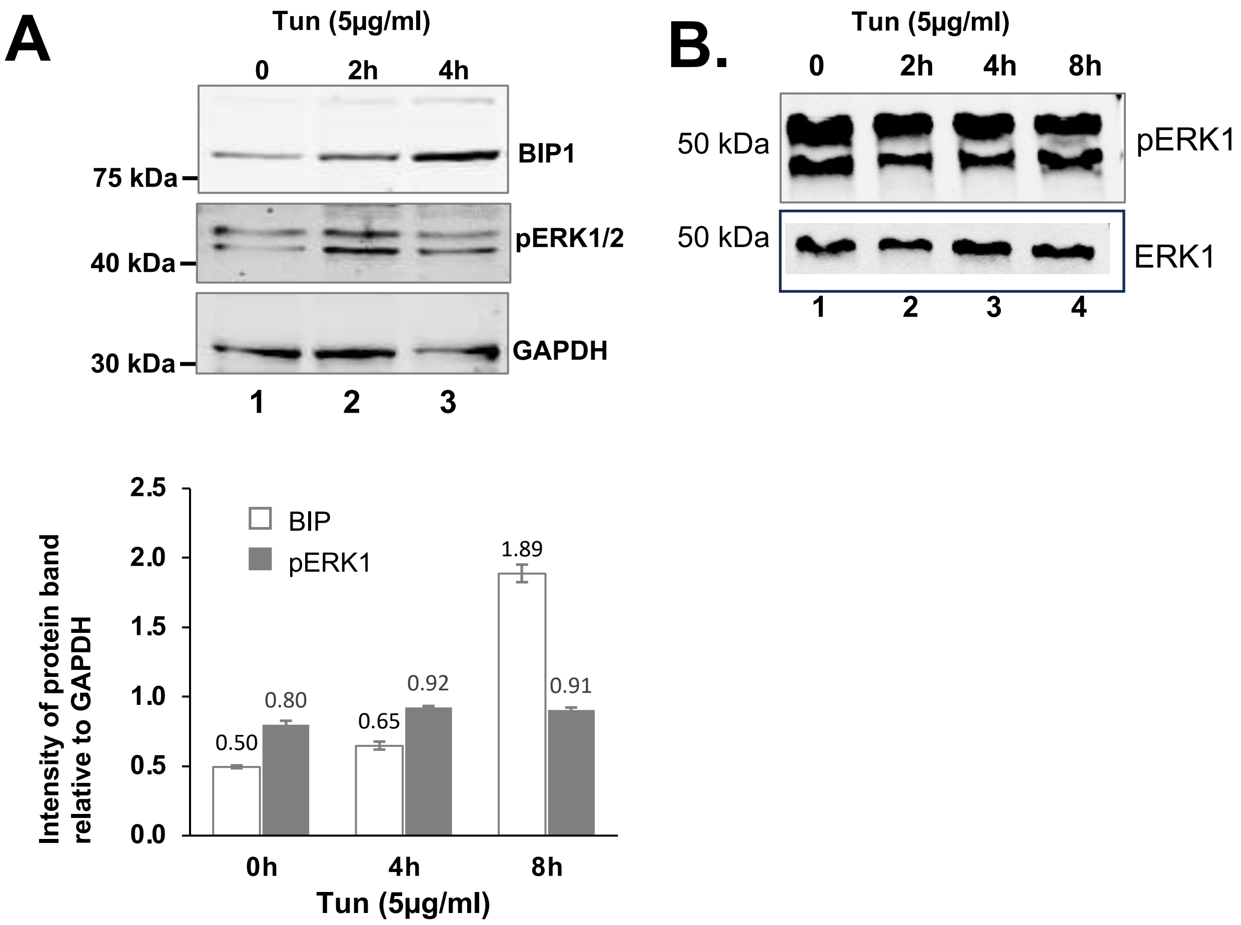

(A) and (B) WCEs were prepared from HEK293FT cells after tunicamycin treatment and subjected to Western blot analysis to detect Bip1, pERK1, ERK1. and GAPDH .
